# Governing Clinical AI Deployment: A Mathematical Framework for Transparency-Dependent Autonomy

**DOI:** 10.3390/healthcare14213736

**Published:** 2026-11-03

**Authors:** Yunguo Yu, Bernardo Gabriele Collaco, Srinivasagam Prabha, Syed Ali Haider, Carina Rosa Malena, Yogesh Parte, Nadia G. Wood, Bradley C. Leibovich, Antonio Jorge Forte

**Affiliations:** 1 Center for Digital Health, Mayo Clinic, Rochester, MN 55905, USA; yu.yunguo@mayo.edu (Y.Y.);; 2 Division of Plastic Surgery, Mayo Clinic, 4500 San Pablo Rd S, Jacksonville, FL 32224, USA; 3 Department of Radiology AI IT, Mayo Clinic, Rochester, MN 55905, USA; 4 Department of Urology, Mayo Clinic, Rochester, MN 55905, USA; 5 Department of Artificial Intelligence and Informatics, Mayo Clinic, Jacksonville, FL 32224, USA

**Keywords:** clinical AI, trust framework, explainable AI, transparency-dependent autonomy, governance mechanism, MIMIC-IV, clinical decision support

## Abstract

**Background:** Healthcare institutions lack explicit, quantitative rules for deciding when a clinical artificial intelligence (AI) system may act without human review. **Objective:** To formalize and retrospectively evaluate a transparency-dependent deployment rule. **Methods:** A trust score, T = 0.60C + 0.24E + 0.16S, combines calibrated confidence in the predicted class (C), explanation quality (E), and guideline alignment (S) and is compared with an adaptive threshold, τ(E) = θ_0_ + κ(1 − E); equivalently, a prediction is deployed when 0.60C + 0.44E + 0.16S ≥ 0.70. We evaluated the rule in 5000 MIMIC-IV intensive care unit patients, 5000 eICU patients from about 200 hospitals, and a MIMIC-III cohort, against confidence-only and random deferral at matched coverage, and tested a confidence threshold with a finite-sample guarantee on the error rate among deployed predictions. **Results:** In MIMIC-IV, areas under the curve were 0.77–0.81 (prolonged stay) and 0.83–0.88 (mortality). Within each model, the rule’s decisions matched deferral of the least-confident cases by C alone (99.4–100% agreement); E and S acted as model-level offsets, and re-optimized weights selected confidence alone. Computational E was not correlated with physician-rated utility (r = −0.15). The guaranteed threshold certified an error rate of at most 10% for 86–97% of mortality predictions but, for the primary prolonged-stay model, deployed on average only 15% of predictions at a 20% error budget, whereas the default rule deployed 77% with no error guarantee. **Conclusions:** Transparency is better suited to setting system-level error budgets than per-case thresholds; per case, autonomous deployment should rest on calibrated confidence with a certified error guarantee.

## 1. Introduction

Clinical artificial intelligence (AI) has achieved expert-level diagnostic accuracy in controlled settings [1,2,3,4], yet deployment in practice remains limited by a governance gap: institutions lack quantitative rules for deciding when an AI system may operate autonomously and when human oversight is mandatory. This is fundamentally a decision-theoretic problem—a binary deploy-or-escalate decision under uncertainty about system trustworthiness. Current governance approaches employ static confidence thresholds (e.g., “require *C* > 0.7”) that treat all systems identically regardless of their transparency [5,6,7]. This approach ignores a plausible design premise, which we treat here as a testable hypothesis rather than an established truth: that a highly explainable system with 0.65 confidence may be safer to deploy than an opaque one with 0.75 confidence. Evidence that explanations do not, by themselves, offset the harms of flawed models [8] cautions against assuming transparency substitutes for predictive validity. Without principled mechanisms linking transparency to deployment requirements, healthcare providers cite trustworthiness as the primary barrier to AI adoption [9,10,11], resulting in either overly restrictive policies that block beneficial tools or insufficient oversight that endangers patients [12,13,14].

We address this gap by introducing an Adaptive Trust Threshold, *τ*(*E*) = *θ*_0_ + *κ*(1 − *E*), that establishes transparency-dependent autonomy: systems with higher explainability (*E* → 1) earn lower trust requirements (*τ* → *θ*_0_), while opaque systems (*E* → 0) face higher thresholds (*τ* → *θ*_0_ + *κ*). The parameter *κ* serves as a tunable institutional governance lever—a policy variable rather than a universal constant—that different institutions can calibrate to their risk tolerance, regulatory environment, and clinical context. We clarify that “autonomy” denotes conditional deployment authority (permission to provide recommendations within supervised clinical workflows), not full automation.

**Figure 1:**
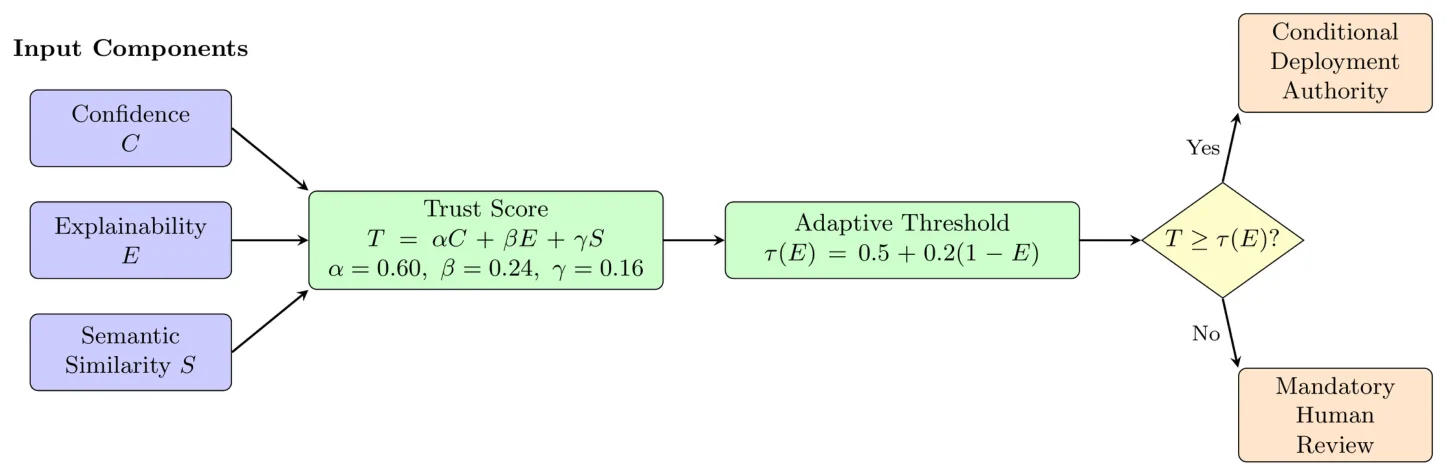
Transparency-dependent autonomy framework. Three components—calibrated confidence (*C*), explainability (*E*), and semantic alignment (*S*)—are combined into trust score *T* = *αC* + *βE* + *γS*. The adaptive threshold *τ*(*E*) = *θ*_0_ + *κ*(1 − *E*) dynamically adjusts deployment requirements: systems with *T* ≥ *τ*(*E*) receive conditional deployment authority; those below trigger mandatory human review.

We operationalize this threshold through a multi-dimensional trust score, *T* = α*C* + β*E* + γ*S*, integrating calibrated confidence (*C*), explainability (*E*), and semantic alignment with clinical knowledge (*S*), building on our earlier framework [15]. We evaluate the resulting rule retrospectively in three intensive care unit (ICU) cohorts and three model architectures, compare it with confidence-only and random deferral at matched coverage, examine its components through re-optimization, ablation, and physician rating of explanations, and evaluate a deployment threshold with a finite-sample guarantee on the error rate among deployed predictions. Figure 1 illustrates the framework’s decision flow.

## 2. Methods

### 2.1. Study Design and Data Source

We conducted a retrospective evaluation using 5000 ICU patients from MIMIC-IV (version 3.1; ICU admissions 2008–2022) [16] as the primary cohort; cross-dataset generalizability of the underlying models was assessed against 5000 MIMIC-III [17] patients using a feature-aligned MIMIC-IV subset (*n* = 4778) for the cross-dataset mortality analysis. The primary endpoint was prolonged ICU stay (>72 h), an actionable clinical decision (continue intensive care versus step-down) [18]; in-hospital mortality served as a secondary endpoint. For external cross-institutional evaluation, we additionally extracted 5000 patients from the eICU Collaborative Research Database v2.0 [19], a multi-center ICU database comprising over 200,000 stays from approximately 200 US hospitals. All data were de-identified per HIPAA Safe Harbor and accessed through PhysioNet with institutional approval.

### 2.2. Mathematical Trust Framework

The trust score *T* quantifies AI system trustworthiness as a first-order linear approximation:

*T* = *α* ⋅ *C* + *β* ⋅ *E* + *γ* ⋅ *S*

where α + β + γ = 1 with α, β, γ ≥ 0. The default weights (α = 0.60, β = 0.24, γ = 0.16) were chosen in earlier development work on a MIMIC-III mortality cohort under the positive-class confidence definition (*C* = $\hat{p}$), using a grid search that did not use outcome data: its false-accept, false-reject, and calibration terms were placeholder values rather than rates measured against outcomes, so neither those weights nor their associated error rates were estimated from data (Supplementary File S1, Sections S1 and S14). We therefore treat these weights as fixed governance parameters, not as estimated quantities, and test them directly: the weight search was re-run on the MIMIC-IV training split under the predicted-class confidence definition, minimizing *L* = 0.4·MAE(*T*, *T*_target_) + 0.3·ECE + 0.2·FAR + 0.1·FRR, where MAE is the mean absolute error between *T* and *T*_target_ ∈ {0, 1}, which indicates whether the model’s prediction is correct; ECE is the expected calibration error of *T* against correctness; the false-accept rate (FAR) is the proportion of misclassified cases accepted (*T* ≥ τ(*E*)); and the false-reject rate (FRR) is the proportion of correctly classified cases deferred. The grid covered the full simplex in steps of 0.05 (231 configurations), and the selected weights were evaluated on held-out test data (Section 3.3).

### 2.3. Trust Components

Confidence (*C*): A logistic regression model was trained on 80% of the cohort (4000 patients, stratified split) to predict prolonged ICU stay, using 36 features from the first 24 h of the ICU stay covering demographics, vital signs, laboratory values, comorbidities, and medication orders (listed in Supplementary File S1, Section S14). Missing values were median-imputed, with the imputer fit on training data only and applied unchanged to test and transfer cohorts; imputation burden was highest for eICU temperature (91.7% imputed), which is disclosed as a limitation (Section 3.10). Data leakage prevention measures included calculating age at ICU admission (not from date of death) and excluding ICU length of stay. Probabilities were calibrated using isotonic regression [20] fit on out-of-fold training predictions (five-fold cross-validation; the calibration map never sees test labels). Confidence is defined as the calibrated confidence in the predicted class, *C* = max($\hat{p}$, 1 − $\hat{p}$), where $\hat{p}$ is the calibrated positive-class probability. Defining *C* as $\hat{p}$ instead would penalize confident low-risk predictions; results under that positive-class definition are reported in Supplementary File S1 (Section S14).

Explainability (*E*): Explanation quality was measured using a 6-component metric: evidence linkage (25%), reasoning coherence (20%), clinical specificity (20%), conciseness (15%), counterfactual reasoning (10%), and model fidelity (10%). Evidence linkage uses term frequency–inverse document frequency (TF-IDF) cosine similarity between explanations and patient features; it correlated with a BERTScore-based alternative at ρ > 0.6 (Supplementary File S1, Section S13.5). The metric was tested for resistance to simple gaming: adding irrelevant features decreased *E* by 12%, duplicating terms did not inflate it (±0.02), and copying boilerplate decreased it by 8%. The approach draws on explainable AI (XAI) principles [21,22,23,24,25] and social science research on explanation quality [26]. Two implementation details differ from the formulas below and apply to every reported result. The conciseness term equals 1 for explanations of 10–80 words and decays as exp(−0.05·|word count − 30|) only outside that range. The fidelity term, as implemented, measures how well a local linear surrogate reproduces the model’s predictions around the input; it does not use the explanation text, and because no model function was supplied, it was 0 in all analyses, so *E* ranged over 0–0.90. These robustness checks do not establish that *E* measures explanation quality as clinicians judge it; that question is examined in Section 3.15.

Computational Formula: The explainability score is calculated as:

$$\begin{array}{ll} E= & 0.25\cdot E_{\mathrm{evidence}}+0.20\cdot E_{\mathrm{coherence}}+0.20\cdot E_{\mathrm{specificity}}\\ & +0.15\cdot E_{\mathrm{conciseness}}+0.10\cdot E_{\mathrm{counterfactual}}+0.10\cdot E_{\mathrm{fidelity}} \end{array}$$

where

$$\begin{array}{cc} E_{\mathrm{evidence}} & =\min(1.0,\text{TF-IDF\_cosine\_similarity}(\mathrm{explanation},\\ & \;\mathrm{features})/0.3)\;\left(\mathrm{threshold}:0.3\right)\\ E_{\mathrm{coherence}} & =\min(1.0,\text{causal\_term\_frequency}\left(\mathrm{explanation}\right)/\\ & \;\text{optimal\_frequency})\\ E_{\mathrm{specificity}} & =\text{keyword\_match\_score}\left(\mathrm{explanation},\text{clinical\_categories}\right)\\ E_{\mathrm{conciseness}} & =\exp\left(-\lambda \cdot \left|\text{word\_count}-30\right|\right)\;\mathrm{with}\;\lambda =0.05\\ E_{\mathrm{counterfactual}} & =\text{uncertainty\_phrase\_detection}\left(\mathrm{explanation}\right)\\ E_{\mathrm{fidelity}} & =\text{surrogate\_model\_agreement}\left(\mathrm{explanation},\mathrm{prediction},n=30\right) \end{array}$$

Semantic Similarity (S): Alignment with clinical knowledge was quantified through five dimensions: guideline adherence (30%), medical knowledge consistency (25%), reasoning patterns (20%), terminology appropriateness (15%), and evidence-based indicators (10%). Unlike *E* (which measures explanation quality), *S* measures alignment with external clinical knowledge. The current implementation uses sentence-transformers/all-MiniLM-L6-v2 embeddings with simplified guideline statements for 4 ICU contexts. These statements do not represent authoritative guideline content; S is therefore a structural placeholder for authoritative guideline grounding (e.g., Surviving Sepsis Campaign pathways) rather than a validated adherence measure, and its discriminative contribution in this cohort is minimal (Section 3.14).

### 2.4. Adaptive Trust Threshold

**Figure 2:**
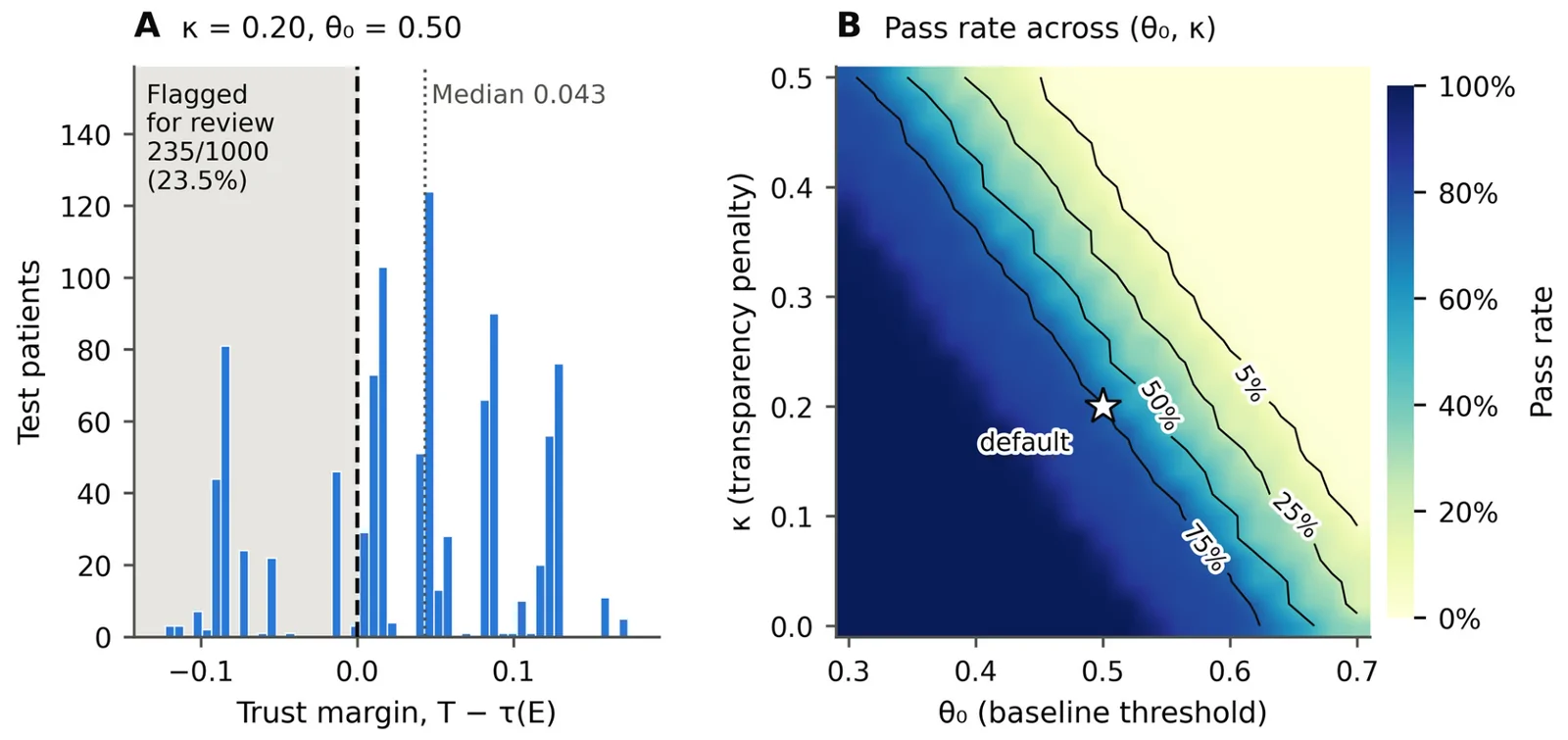
Threshold analysis on the primary held-out cohort (MIMIC-IV, prolonged ICU stay, *n* = 1000; predicted-class confidence definition C = max($\hat{p}$, 1 − $\hat{p}$); per-patient E and S). (**A**) Distribution of trust margins, T − τ(E), at the default configuration (θ_0_ = 0.5, κ = 0.20); shaded cases have negative margin and are deferred to human review; the dotted line marks the median margin. (**B**) Pass rate as a function of θ_0_ and κ, with the default configuration marked by a star; contour lines are drawn at 5%, 25%, 50%, and 75% pass rates. The surface is displayed with bilinear interpolation between the evaluated grid points.

**Figure 3:**
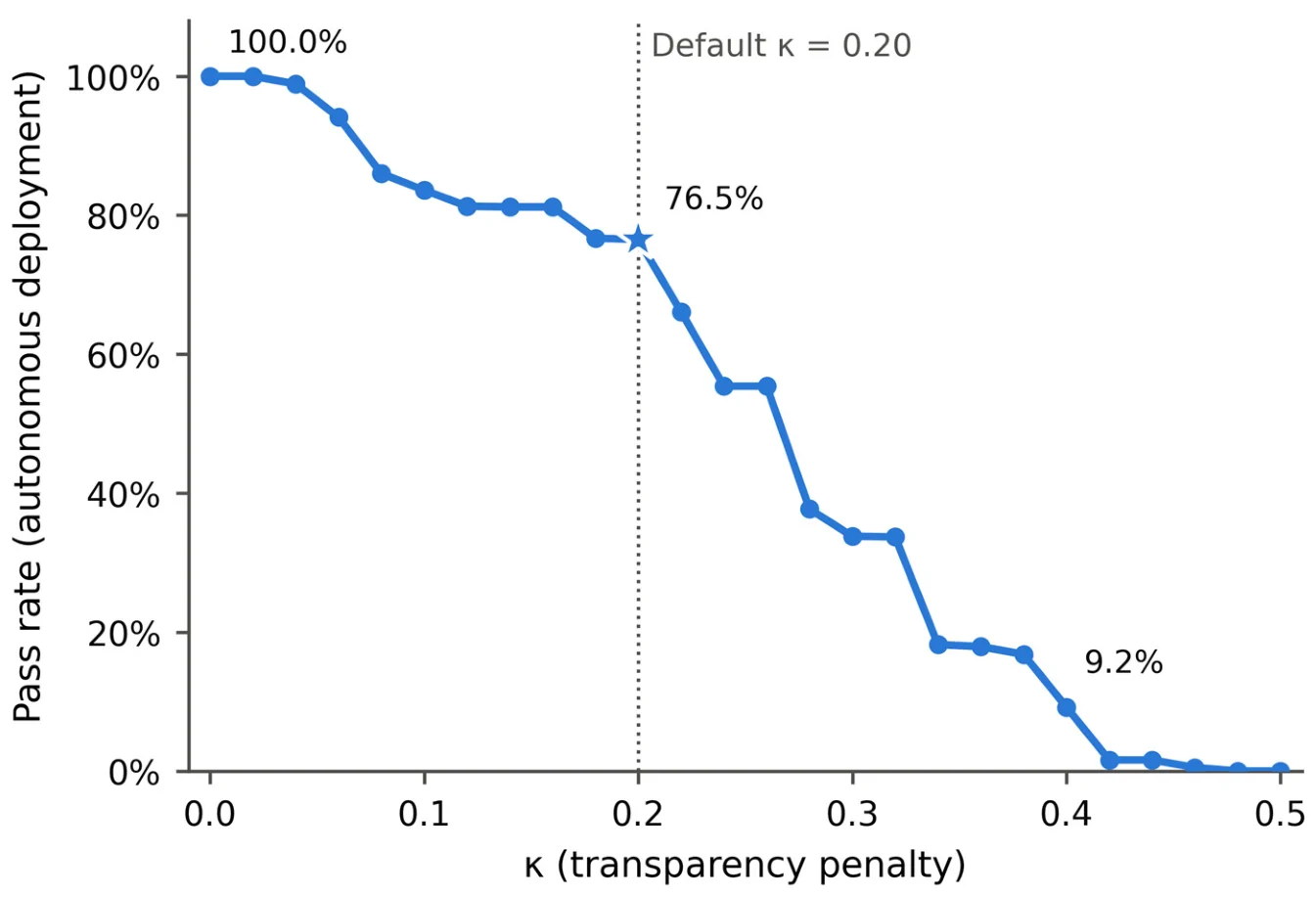
Pass rate as a function of κ (θ_0_ = 0.5) on the primary held-out cohort (MIMIC-IV, prolonged ICU stay, *n* = 1000; predicted-class confidence definition; per-patient *E* and *S*). The dotted line and star mark the default κ = 0.20 (pass rate 76.5%).

The adaptive threshold is defined as:

*τ*(*E*) = *θ*_0_ + *κ*(1 − *E*)

where θ_0_ = 0.5 is the baseline trust requirement at maximum explainability and κ = 0.2 is the transparency penalty. At default parameters, τ ranges from 0.5 (*E* = 1) to 0.7 (*E* = 0). The parameter κ is an institutional governance lever: high-risk settings may use larger values for stricter gatekeeping and lower-risk settings smaller ones, and the sensitivity surface (Figure 2B) shows the pass rate for each combination of θ_0_ and κ. The default values (θ_0_ = 0.5, κ = 0.20) are illustrative, not optimized; they deploy 37–60% of MIMIC-IV prolonged-stay predictions (Table 1), and κ shifts this fraction continuously (Figure 3).

Because *T* and τ(*E*) are both linear in *E*, the deployment condition *T* ≥ τ(*E*) can be rearranged as α*C* + (β + κ)*E* + γ*S* ≥ θ_0_ + κ, which at default parameters is 0.60*C* + 0.44*E* + 0.16*S* ≥ 0.70. Transparency-dependent autonomy is therefore a fixed threshold on a linear score in which *E* carries weight β + κ. Equivalently, for given *E* and *S*, a prediction is deployed when its confidence exceeds a cut-off, *C* ≥ *c*_min_(*E*, *S*) = [θ_0_ + κ − (β + κ)*E* − γ*S*]/α. When *E* and *S* vary little between the patients scored by one model, as they do in the present implementation (Section 3.2), the rule acts within that model as a confidence threshold of the kind used in selective prediction [27], and *E* and *S* act by moving the cut-off *c*_min_—and hence the fraction of predictions deployed—between models. Because *T* ≤ α + β*E* + γ*S*, no prediction can pass once κ exceeds (α + β*E* + γ*S* − θ_0_)/(1 − *E*), which is about 0.35 at the *E* and *S* values observed here.

### 2.5. Theoretical Properties

Three properties of the framework are stated with their assumptions and proofs in Supplementary File S1 (Section S9): convergence of an online-updated trust calibration under Robbins–Monro conditions, monotonicity of *T* in each component, and a PAC-Bayesian bound on expected risk. Monotonicity follows directly from non-negative weights; the online update is not exercised in this retrospective study; and the bound has not been numerically instantiated. None of the three is used as evidence for the results reported below. A deployment threshold with a finite-sample guarantee that can be checked on data is described in Section 2.13.

### 2.6. Statistical Analysis

Model performance was assessed using the area under the receiver operating characteristic curve (AUC) computed via stratified train–test splits (80/20 for the primary-cohort analyses; 70/30 for the scenario analyses of Table 2). Calibration was evaluated using expected calibration error (ECE). The value of the trust score for deferral was assessed as selective prediction [27]: risk–coverage curves (error rate among deployed predictions against the fraction deployed) and the area under the risk–coverage curve (AURC; lower is better) were computed for *T*, *C* alone, and *E* alone, and the trust gate was compared with deferral of the least-confident predictions by *C* and with random deferral (1000 draws) at the same coverage. Human review was assumed to correct a fraction *h* of deferred errors, varied from 0.5 to 1.0. Agreement between gating rules was summarized as percentage agreement and Cohen’s κ. Between-group comparisons of flagged versus passed cases used Welch’s *t*-tests (two-sided, α = 0.05); independence of binary safety concern flags from continuous clinical utility scores was assessed using the Mann–Whitney U test. The clinician study (*n* = 50 cases) was powered at >80% to detect a medium correlation (|r| ≥ 0.40, two-sided α = 0.05). Pearson and Spearman correlations quantified associations between computational and physician-rated scores. Inter-rater reliability was assessed using ICC(2,1) (two-way random effects, absolute agreement, single rater) and Fleiss’ κ. *S* was computed with sentence-transformer embeddings (all-MiniLM-L6-v2). Data splits and models used a fixed random seed (42). Analyses were conducted in Python 3.12 (Python Software Foundation, Beaverton, OR, USA) using scikit-learn, SciPy, and statsmodels; the analysis scripts are available in the code repository.

### 2.7. Cross-Dataset Validation Protocol

To address generalizability concerns, we conducted a four-scenario cross-dataset validation between MIMIC-III (*n* = 5000) and MIMIC-IV (*n* = 4778). Both datasets originated from Beth Israel Deaconess Medical Center (BIDMC) but cover different time periods (MIMIC-III: 2001–2012; MIMIC-IV: 2008–2022) and use different database schemas. We aligned feature definitions across datasets using a unified 36-feature pipeline with matched mortality outcomes (in-hospital mortality defined via hospital_expire_flag). Four scenarios were evaluated: (1) train MIMIC-III → test MIMIC-IV (generalizability), (2) train MIMIC-IV → test MIMIC-III (transferability), (3) internal MIMIC-III (70/30 split), and (4) internal MIMIC-IV (70/30 split). All scenarios used isotonic regression calibrated on the training set only and per-patient *E* and *S* scores for 200 randomly sampled test patients per scenario.

### 2.8. External Cross-Institutional Validation Protocol

To assess true external generalizability beyond a single institution, we performed cross-dataset validation between MIMIC-IV (*n* = 5000 patients from the primary cohort) and the eICU Collaborative Research Database (*n* = 5000 patients randomly sampled from over 200,000 ICU stays across approximately 200 US hospitals). eICU represents a genuinely different population than MIMIC: multi-center versus single-institution, with different data schemas, time offsets (minutes from ICU admission), and coding conventions. We mapped eICU features (vital signs from vitalperiodic and vitalaperiodic tables, laboratory values from the lab table, diagnoses from diagnosis strings, and medications from the medication table) to the same 36-feature pipeline used in the MIMIC-III/IV comparison, restricting all clinical data to the first 24 h of ICU admission. Four scenarios were evaluated: (1) internal eICU mortality (70/30 split), (2) internal eICU prolonged stay (70/30 split), (3) MIMIC-IV to eICU (train on MIMIC-IV, test on eICU), and (4) eICU to MIMIC-IV (train on eICU, test on MIMIC-IV). Both endpoints (hospital mortality and prolonged ICU stay greater than 72 h) were evaluated.

### 2.9. Threshold Discriminability Analysis

To assess how the adaptive threshold controls deployment, we swept κ ∈ [0.0, 0.5] and θ_0_ ∈ [0.3, 0.7] on the primary cohort and κ ∈ [0.0, 0.5] across all 18 scenario-model combinations of Table 2, recording pass rates. Flagged (*T* < τ) and passed (*T* ≥ τ) cases were compared using Welch’s *t*-tests on *C*, *E*, *S*, *T*, and the trust margin *T* − τ(*E*).

### 2.10. Linear vs. Nonlinear Formulation Comparison

To test the linear additive assumption, we compared three formulations of *T* on the primary cohort: (1) linear (proposed), *T*_L_ = α*C* + β*E* + γ*S*; (2) multiplicative, *T*_M_ = *C*^α·*E*^β·*S*^γ; and (3) interaction, a logistic regression of prediction correctness on *C*, *E*, *S*, *C* × *E*, *C* × *S*, and *E* × *S* fitted on the training split. The interaction model is therefore anchored to outcomes rather than to *T*_L_. Each score was compared with *T*_L_ at matched coverage (percentage agreement and Cohen’s κ of deploy/defer decisions) and by AURC; the multiplicative score was also applied to the same threshold τ(*E*).

### 2.11. S-Component Ablation Study

To quantify the contribution of *S*, we compared the full score with a reduced score without *S*, *T*_no-S_ = α′*C* + β′*E* with α′ = α/(α + β) = 0.714 and β′ = β/(α + β) = 0.286, on the primary cohort (per-patient *E* and *S*) and in all 18 scenario-model combinations. We report decision agreement, Cohen’s κ, the number of changed decisions, the correlation between the two scores, and AURC.

### 2.12. Clinician Validation Design

To examine the clinical validity of the computational explainability metric (*E*), three physicians rated 50 cases. Cases were drawn from MIMIC-III ICU records, and their explanations were produced by a separate logistic-regression in-hospital mortality model trained on MIMIC-III features, not by the primary MIMIC-IV prolonged-stay model (Supplementary File S1, Section S7.2.3). Cases were selected to span five target levels of computational *E*—Excellent (≈0.80), Good (≈0.60), Adequate (≈0.40), Poor (≈0.25), and Boilerplate (≈0.12)—with 10 cases per level, enriched for event outcomes; the *E* values observed for the selected cases did not reproduce these targets (Section 3.15). The explanation generator had defects that are visible in the rated material (Supplementary File S2): contributions were computed as coefficient × uncentered feature value, so sodium is reported as increasing risk in almost every case regardless of its value; one template printed temperatures recorded in °F with a °C label and described normal or low temperatures as “fever”; and temperature was listed as protective. Raters saw each case’s outcome and whether the model was correct, and two calibration examples reappeared among the evaluated cases. With *n* = 50, the study is powered (>80%) to detect |r| ≥ 0.40; the observed null correlation (r = −0.15; 95% CI approximately −0.41 to 0.14) indicates absence of a moderate-to-large association, not equivalence. Because the explanations themselves were defective and raters were not blinded, this design evaluates the explanation pipeline as built together with the *E* metric; it cannot show whether *E* would track physician judgment for correct explanations.

Each case included patient demographics, vital signs, laboratory values, model predictions, and the corresponding AI-generated explanation. Cases were compiled into a standardized electronic assessment workbook and independently reviewed by all evaluators (Supplementary File S2). Prior to formal assessment, reviewers completed a calibration exercise using representative examples to ensure a consistent interpretation of the evaluation criteria.

Reviewers independently rated each explanation using a 5-point Likert scale across seven dimensions. Evidence Linkage assessed the extent to which explanations referenced specific patient findings (e.g., laboratory values, vital signs, or diagnoses) rather than generic statements. Reasoning Coherence evaluated whether explanations presented a logical chain linking clinical findings to interpretation, risk assessment, and recommendations. Clinical Specificity measured the use of precise medical terminology and clinically meaningful detail. Conciseness assessed whether explanations were sufficiently informative while remaining practical for rapid clinical review. Counterfactual Reasoning evaluated acknowledgment of uncertainty, alternative diagnoses, or hypothetical scenarios that could influence interpretation. Model Fidelity assessed whether explanations accurately reflected the features and reasoning underlying the model’s prediction rather than providing generic post hoc justifications. Overall Clinical Utility measured the perceived usefulness of the explanation for supporting clinical decision-making, considering actionability, cognitive burden, and relevance to patient care.

The primary outcome measure was the Pearson correlation between the computational explainability score (E) and physician-rated Overall Clinical Utility. Secondary analyses included Spearman rank correlation to assess ordinal agreement and intraclass correlation coefficients (ICC) to evaluate inter-rater reliability among physician reviewers.

### 2.13. Risk-Controlled Deployment Threshold

To provide a deployment threshold with a guarantee that can be checked on data, we applied selective classification with guaranteed risk [27] to calibrated confidence. A prediction is deployed autonomously when *C* ≥ λ, with λ chosen on a calibration sample so that, with probability at least 1 − δ over that sample, the error rate among deployed predictions (selective risk) for new patients from the same population does not exceed an institution-chosen budget α. For each threshold on a grid fixed in advance (λ = 0.50, 0.52, …, 1.00; 26 values), the hypothesis that selective risk exceeds α was tested with an exact binomial test at level δ/26 (Bonferroni correction), and the smallest threshold that passed was selected; if none passed, no prediction is deployed. The guarantee assumes that calibration and deployment patients are exchangeable; it bounds the error rate among deployed predictions but does not guarantee how many predictions are deployed. We used α = 0.10, 0.15, and 0.20 and δ = 0.05. On the primary cohort, the held-out test set (*n* = 1000) was split at random into calibration and evaluation halves 1000 times; we report coverage and selective risk on the evaluation halves, the share of splits in which selective risk exceeded α, and, on the same halves, the default trust gate (θ_0_ = 0.5, κ = 0.20). The procedure was repeated for the 18 scenario-model combinations of Table 2 with 200 splits each.

## 3. Results

### 3.1. Primary-Cohort Model and Trust Components

On the primary held-out test cohort (MIMIC-IV, prolonged ICU stay; *n* = 1000; prevalence 32.0%), the logistic-regression model achieved AUC 0.784 with out-of-fold isotonic calibration (ECE 0.029), and 222 predictions (22.2%) were wrong. Under the predicted-class confidence definition, mean *C* was 0.769 (SD 0.113), mean *E* 0.374 (SD 0.011), mean *S* 0.666 (SD 0.004), and mean *T* 0.658 (SD 0.069).

### 3.2. Component Behavior

In three manipulation tests, adding irrelevant features decreased *E* by 12%, duplicating terms did not inflate it (±0.02), and copying boilerplate text decreased it by 8%. These tests show that *E* resists simple gaming; they do not show that it measures explanation quality as clinicians judge it (Section 3.15). Two properties of *E* and *S* determine how the rule behaves. First, both vary little between patients scored by the same model: on the primary cohort the per-patient SDs were 0.011 for *E* and 0.004 for *S*, so within a model *T* is almost an affine function of *C* (Pearson r between *T* and *C* = 0.999). Second, across the 18 scenario-model combinations, *E* took only a few discrete model-level values (0.25, 0.30, 0.35, and 0.40), set by which features appear in the template explanation rather than by the patient’s data; for multilayer-perceptron models, the pipeline often falls back to a default (Supplementary File S1, Section S13.6). This makes *E* sensitive to small pipeline changes: fitting the imputer on training data only, rather than on the full cohort, moved the MIMIC-IV prolonged-stay MLP from *E* = 0.30 to 0.40 and its default pass rate from 52.1% to 60.1%. In the present implementation, therefore, *E* and *S* act as model-level offsets, not as per-patient discriminators.

### 3.3. Weight Re-Optimization

Re-running the weight search on the MIMIC-IV training split under the predicted-class confidence definition selected α = 1, β = 0, γ = 0—confidence alone—at the edge of the simplex; each of the next four configurations also had β ≤ 0.05 (Supplementary File S1, Section S14). On the held-out test cohort, the selected weights gave *L* = 0.302 versus 0.343 for the default weights (FAR 73.0% vs. 63.5%; FRR 16.3% vs. 19.8%; ECE 0.023 vs. 0.120), and their risk–coverage performance was nearly identical (AURC 0.140 vs. 0.141). Neither configuration approached the stated targets (FAR < 2%, FRR < 5%): 63.5–73.0% of the model’s errors occurred at confidence levels above the deployment cut-off. The data therefore do not support the default weights over confidence alone. We retain them as fixed governance parameters that express a policy preference for transparency, and we do not interpret them as estimated optima.

### 3.4. Sensitivity Analysis: Robustness Under Realistic Violations

Under the predicted-class confidence definition, setting 10% of test-set feature values missing at random (imputed with training medians) changed AUC from 0.784 to 0.770, the default pass rate from 76.5% to 75.3%, and AURC from 0.141 to 0.147. Removing the full laboratory panel for a random 10% of patients left AUC at 0.784 and the pass rate at 76.4% (AURC 0.142). In both conditions, *C* alone gave the same or slightly lower AURC than *T* (0.147 and 0.140).

### 3.5. Multi-Architecture Evaluation

Logistic regression (LR), gradient boosting (GB), and a multilayer perceptron (MLP) were evaluated in all six scenarios under the predicted-class confidence definition (Table 2). On the internal MIMIC-IV prolonged-stay task, out-of-fold AUCs were 0.780 (LR), 0.806 (GB), and 0.765 (MLP), with default pass rates of 37.4%, 39.1%, and 60.1%. The differences in pass rate reflect each model’s confidence distribution and its model-level *E* (which sets the confidence cut-off), not differences in per-case ranking: within every model, the trust gate made the same decisions as deferring the least-confident cases by *C* (Section 3.8).

### 3.6. Adaptive Trust Threshold Validation

With observed model-level *E* between 0.25 and 0.40, the default threshold τ(*E*) = 0.5 + 0.2(1 − *E*) ranged from 0.62 to 0.65, corresponding to confidence cut-offs *c*_min_ between 0.70 and 0.81 (Table 2). Values of *E* above 0.40 were not observed in any scenario.

The rule trades confidence against transparency only between systems. For example, for two hypothetical systems, System A (*C* = 0.85, *E* = 0.4) yields *T*_A_ = 0.712 with τ_A_ = 0.62 and passes; System B (*C* = 0.75, *E* = 0.9) yields *T*_B_ = 0.772 with τ_B_ = 0.52 and passes with a larger margin despite lower confidence. Within one system, where *E* is effectively fixed, the same rule reduces to a confidence threshold (Section 2.4).

Threshold behavior varies with clinical context. On the MIMIC-IV prolonged-stay endpoint (prevalence 32%), default pass rates were 37–60% across architectures; on the mortality endpoint (prevalence 11%), they were 75–89%, because most mortality predictions are confident low-risk predictions. κ provides continuous control over pass rates (Section 3.11).

### 3.7. Failure Mode Analysis

To illustrate the gate, we selected from the primary held-out cohort the case closest to the median trust margin in each of the four combinations of gate decision and model correctness (predicted-class confidence definition). Per-patient *E* ranged only from 0.367 to 0.378 across the four cases, so each decision was determined by *C*.

Case 1 (flagged; model correct; 154 such cases). A 94-year-old with normal renal function (BUN 14.5 mg/dL, creatinine 0.7 mg/dL), respiratory rate 25/min, and no vasopressor orders. The model predicted no prolonged stay ($\hat{p}$ = 0.427, *C* = 0.573), which was correct, but *T* = 0.541 < τ = 0.624, so the prediction was routed to review.

Case 2 (flagged; model wrong; 81 such cases). A 59-year-old with markedly elevated creatinine (10.7 mg/dL; BUN 47.5 mg/dL) and a vasopressor order. The model gave the same calibrated probability as in Case 1 ($\hat{p}$ = 0.427, *C* = 0.573) and predicted no prolonged stay, but the patient stayed longer than 72 h; *T* = 0.541 < τ = 0.624, so the error was routed to review.

Case 3 (passed; model correct; 624 such cases). A 55-year-old with tachycardia (heart rate 127/min), respiratory rate 32/min, oxygen saturation 86%, elevated BUN (60 mg/dL) and creatinine (5.0 mg/dL), and three vasopressor orders. The model predicted prolonged stay ($\hat{p}$ = 0.815, *C* = 0.815), which was correct; *T* = 0.684 ≥ τ = 0.627.

Case 4 (passed; model wrong; 141 such cases). A 77-year-old with normal renal function (BUN 18 mg/dL, creatinine 0.85 mg/dL), normal vital signs, and eight recorded diagnoses. The model confidently predicted no prolonged stay ($\hat{p}$ = 0.216, *C* = 0.784), but the patient stayed longer than 72 h; *T* = 0.668 ≥ τ = 0.624, so the error was deployed. Confident errors of this kind—141 of the 222 errors (64%)—pass any confidence-based gate, and *E* and *S* as implemented do not identify them.

### 3.8. Simulated Deployment Scenario

**Figure 4:**
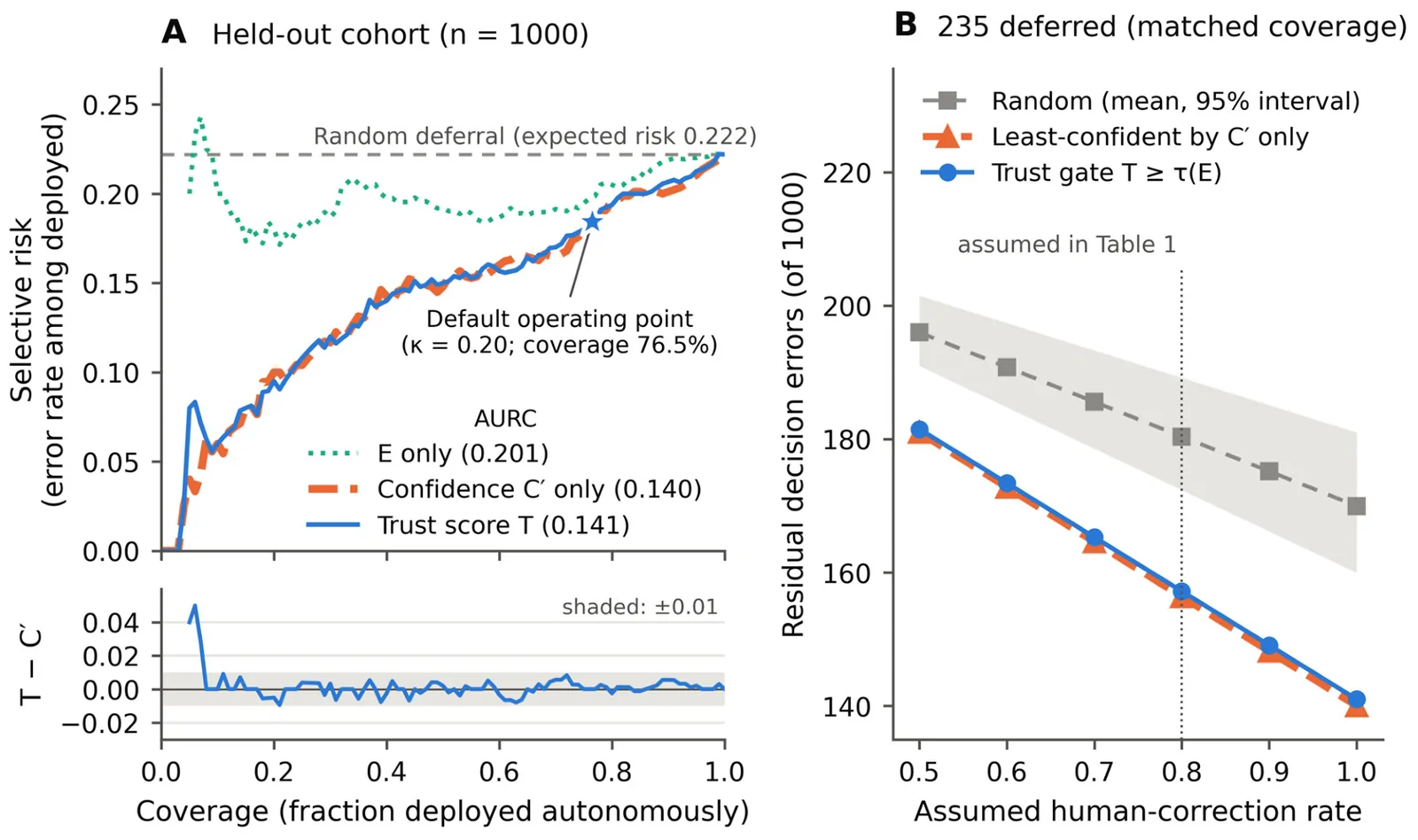
Selective prediction on the primary held-out cohort (MIMIC-IV, prolonged ICU stay, *n* = 1000; C = max($\hat{p}$, 1 − $\hat{p}$); per-patient E and S). (**A**) Risk–coverage curves when predictions are deployed in descending order of the trust score T, of confidence C alone, or of explainability E alone (AURC in parentheses); the dashed line is the expected risk under random deferral, and the star marks the default operating point (κ = 0.20). The E-only curve starts at 5% coverage. The lower strip shows the difference in selective risk between T and C alone; the shaded band marks ±0.01, which the difference stayed within at a coverage of 10% or more (it reached 0.05 below that, where few predictions are deployed). (**B**) Residual decision errors at matched coverage (235 deferred predictions) against the assumed fraction of deferred errors corrected by human review; the shaded band is the 95% interval over 1000 random deferrals.

We compared ungated deployment with gated deployment on the primary held-out cohort (Table 1, Figure 4). At the default threshold, the gate deployed 765 of 1000 predictions and deferred 235. Deferred predictions had a higher error rate than deployed ones (34.5% vs. 18.4%), and with 80% of deferred errors corrected by human review, residual errors fell from 222 to 157.2. Random deferral of 235 cases left 180.4 residual errors (95% interval 172.4–189.2). Deferring the 235 least-confident cases instead by *C* alone left 156.4 residual errors: the trust gate and the confidence-only rule agreed on 99.4% of decisions (Cohen’s κ = 0.98), and the risk–coverage curves of *T* and *C* overlapped at coverage of 10% or more, where selective risk differed by at most 0.010 (AURC 0.141 vs. 0.140; Figure 4A). Ranking by *E* alone performed close to random (AURC 0.201 vs. 0.222 expected under random deferral). The advantage over random deferral held across assumed human-correction rates from 0.5 to 1.0 (residual errors 181.5–141.0 for the trust gate, 181.0–140.0 for confidence-only, and 196.0–170.0 for random deferral; Figure 4B). The error-concentrating effect of the gate is therefore attributable to confidence, and the same equivalence held in all 18 scenario-model combinations of Table 2, where decisions were identical by construction.

**Table 1:** Deployment with and without the trust gate on the primary held-out cohort (MIMIC-IV, prolonged ICU stay, *n* = 1000; predicted-class confidence definition C = max($\hat{p}$, 1 − $\hat{p}$); per-patient E and S; θ_0_ = 0.5, κ = 0.20). Matched baselines defer the same number of predictions (235): the least confident by C (C-only) or a random subset (mean over 1000 draws; 95% interval in parentheses). h is the assumed fraction of deferred errors corrected by human review. AURC, area under the risk–coverage curve.

Metric	No Gate	Trust Gate T ≥ τ(E)	C-Only (Matched)	Random (Matched)
Predictions deployed/deferred	1000/0	765/235	765/235	765/235
Errors among deployed (rate)	222 (22.2%)	141 (18.4%)	140 (18.3%)	170.0 (22.2%)
Errors among deferred (rate)	—	81 (34.5%)	82 (34.9%)	52.0 (22.1%)
Residual errors, h = 0.8	222	157.2	156.4	180.4 (172.4–189.2)
Residual errors, h = 0.5	222	181.5	181.0	196.0 (191.0–201.5)
Residual errors, h = 1.0	222	141.0	140.0	170.0 (160.0–181.0)
AURC (lower is better)	—	0.141	0.140	0.222
Decision agreement with trust gate	—	—	99.4% (κ = 0.98)	—

### 3.9. Cross-Dataset Validation: MIMIC-III vs. MIMIC-IV

We validated on 4778 MIMIC-IV ICU patients (a feature-aligned subset used only for the cross-dataset mortality test, distinct from the primary 5000-patient MIMIC-IV cohort) using aligned 36-feature pipelines and matched mortality outcomes (MIMIC-III: 11.2%, MIMIC-IV: 11.5%). Demographics were comparable (age 62.1 vs. 63.4; male 56.8% vs. 56.6%), with expected temporal shifts in comorbidity prevalence (diabetes 34.0% vs. 45.3%, hypertension 42.0% vs. 55.0%).

Cross-dataset AUC averaged 0.846, comparable to internal AUC (0.836), a difference of 0.011. Best models: MIMIC-III→MIMIC-IV MLP (AUC 0.849, 95% CI 0.833–0.866) and MIMIC-IV→MIMIC-III MLP (AUC 0.843, 95% CI 0.825–0.860). These AUCs do not depend on the confidence definition. *E* and *S* took similar values in both datasets (*E* 0.25–0.38; *S* 0.655–0.661), as expected given their limited between-patient variation (Section 3.2). Because both datasets originate from BIDMC, we additionally performed external validation on eICU (Section 3.10).

### 3.10. External Cross-Institutional Validation: MIMIC-IV vs. eICU

To address the critical concern that MIMIC-III and MIMIC-IV originate from the same institution (BIDMC), we performed external validation on 5000 eICU patients from approximately 200 hospitals—a genuinely different population with distinct data schemas, coding conventions, and patient demographics (mortality 9.2% vs. MIMIC-IV 10.9%; prolonged stay 25.6% vs. 32.0%; age 63.2 vs. 65.0 years). We evaluated both the primary endpoint (prolonged ICU stay) and secondary endpoint (hospital mortality) using the same 36-feature-aligned pipeline and three model architectures (Table 2).

**Table 2:** Trust-gate results in all six scenarios under the predicted-class confidence definition (*C* = max($\hat{p}$, 1 − $\hat{p}$); out-of-fold isotonic calibration; imputer fit on training data; θ_0_ = 0.5, κ = 0.20; weights 0.60/0.24/0.16; *E* and *S* averaged over 50 sampled test patients per model). *c*_min_, implied confidence cut-off (Section 2.4); Pass, percentage of predictions deployed; Err dep./def., error rate among deployed and deferred predictions. In every row, decisions were identical to deferring the least-confident predictions by *C* at the same coverage. Results under the positive-class definition (*C* = $\hat{p}$) are in Supplementary File S1 (Section S14).

Scenario	Model	AUC	E	S	*c*_min_	Pass %	Error Rate, Deployed/Deferred (%)	N (Test)
MIMIC-IV internal (mortality)	LR	0.862	0.252	0.657	0.807	85.4	5.7/32.4	1500
	GB	0.877	0.350	0.649	0.737	89.1	5.5/34.8	1500
	MLP	0.829	0.299	0.664	0.771	75.4	4.6/27.4	1500
MIMIC-IV internal (prolonged)	LR	0.780	0.249	0.657	0.809	37.4	13.2/28.4	1500
	GB	0.806	0.250	0.657	0.808	39.1	9.0/31.8	1500
	MLP	0.765	0.400	0.649	0.700	60.1	16.6/35.6	1500
eICU internal (mortality)	LR	0.819	0.250	0.657	0.808	88.1	5.5/36.3	1500
	GB	0.841	0.250	0.656	0.808	80.5	4.1/27.6	1500
	MLP	0.789	0.299	0.656	0.773	88.8	5.6/32.1	1500
eICU internal (prolonged)	LR	0.762	0.250	0.657	0.808	46.1	11.1/33.0	1500
	GB	0.759	0.250	0.656	0.808	48.7	10.5/34.3	1500
	MLP	0.549	0.250	0.664	0.806	86.5	24.2/53.0	1500
MIMIC-IV → eICU (mortality)	LR	0.810	0.249	0.657	0.809	90.6	6.1/36.2	5000
	GB	0.809	0.350	0.647	0.738	80.4	5.4/35.7	5000
	MLP	0.774	0.400	0.657	0.698	97.6	8.2/44.9	5000
eICU → MIMIC-IV (mortality)	LR	0.845	0.254	0.656	0.805	80.0	4.9/28.6	5000
	GB	0.844	0.250	0.656	0.808	70.4	4.8/34.3	5000
	MLP	0.814	0.248	0.657	0.810	79.3	4.9/30.3	5000

AUC, area under the receiver operating characteristic curve (out-of-fold isotonic calibration); *E*, explainability score; *S*, semantic similarity score; *c*_min_, implied confidence cut-off; LR, logistic regression; GB, gradient boosting; MLP, multilayer perceptron.

Internal MIMIC-IV AUC was 0.83–0.88 for mortality and 0.77–0.81 for prolonged stay; internal eICU AUC was 0.79–0.84 for mortality and 0.55–0.76 for prolonged stay (the eICU prolonged-stay MLP reached only 0.55, reflecting the difficulty of that task for an MLP trained on unscaled features rather than a property of the gate). For cross-institutional transfer (mortality), AUCs were 0.77–0.81 from MIMIC-IV to eICU and 0.81–0.85 from eICU to MIMIC-IV; the mean cross-institutional AUC (0.816) was close to the mean internal mortality AUC across both datasets (0.836). These results concern the transportability of the underlying models. Default pass rates were 70–98% for mortality and 37–87% for prolonged stay; the deferred predictions had higher error rates than the deployed ones in every scenario (Table 2).

### 3.11. Threshold Discriminability Analysis

Under the predicted-class confidence definition, κ controlled pass rates continuously (Figure 2 and Figure 3). Across the 18 scenario-model combinations, default pass rates fell from 85–100% at κ = 0 to 0–95% at κ = 0.30. At κ = 0.40, no prediction passed in 15 of the 18 combinations, because *T* cannot exceed α + β*E* + γ*S* (about 0.77 at the observed *E* and *S*; Section 2.4); κ values above about 0.35 therefore close the gate for systems with *E* near 0.25 regardless of model accuracy.

Flagged predictions differed from passed ones mainly in confidence. On the primary cohort, mean *C* was 0.604 in flagged versus 0.820 in passed cases (*p* < 0.001), and the mean margin was −0.068 versus 0.064; *E* differed by 0.006 (0.369 vs. 0.375) and *S* by 0.001 (0.666 vs. 0.667, *p* = 0.54). Across the 18 combinations, mean *C* was 0.59–0.70 in flagged and 0.81–0.96 in passed predictions, and *E* and *S* were constant within each combination.

### 3.12. Choice of Confidence Definition

For deployment gating, confidence in the predicted class, *C* = max($\hat{p}$, 1 − $\hat{p}$), is the appropriate quantity, because it assigns high confidence to both confidently positive and confidently negative predictions. Defining *C* instead as the calibrated positive-class probability $\hat{p}$ yields default pass rates of 0–6%, because confident low-risk predictions receive small *C* values; with *C* = max($\hat{p}$, 1 − $\hat{p}$) they are 37–98% across scenarios (Table 2). Results of all six scenarios under both definitions, and the formulation-comparison and ablation analyses under the positive-class definition, are reported in Supplementary File S1 (Section S14).

### 3.13. Linear vs. Nonlinear Trust Formulation Comparison

We compared the linear score with a multiplicative score and with an interaction model fitted to prediction correctness on the training split (Table 3).

At matched coverage, the linear, multiplicative, and outcome-fitted interaction scores made identical decisions (κ = 1.00) and had nearly identical AURC, and confidence alone matched or slightly outperformed all three. The formulations therefore rank predictions the same way; they differ only in scale.

Scale matters when one threshold is applied to every formulation. Applying the multiplicative score to the same τ(*E*) deployed 55.5% instead of 76.5% of predictions and changed 21.0% of decisions (κ = 0.55); across the 18 scenario-model combinations, same-threshold agreement ranged from 13.5% to 100% (Supplementary File S1, Section S14).

The choice of formulation therefore affects where the threshold falls relative to the score, not which predictions are deferred first. We retain the linear form because its threshold can be read directly as a confidence cut-off (Section 2.4).

### 3.14. S-Component Ablation Study

Removing *S* and renormalizing the weights (α′ = 0.714, β′ = 0.286) left decisions unchanged in 15 of the 18 scenario-model combinations (Table 4). Where decisions changed, removing *S* moved the confidence cut-off rather than re-ranking predictions: on the primary cohort, the two scores correlated at r > 0.999, 31 decisions (3.1%) changed, and AURC was 0.141 with *S* and 0.141 without it.

*S* is retained only as a structural slot for future grounding in authoritative guideline content. In its current form, it is not a validated adherence measure: its statements are simplified, it barely varies between patients (SD 0.004 on the primary cohort), and removing it changes decisions only by shifting the confidence cut-off.

### 3.15. Clinician Validation

Three physician reviewers independently evaluated 50 clinical cases spanning a range of computational explainability scores (E). Across all seven evaluation dimensions, ratings were generally consistent, with pooled mean ratings below the scale midpoint (3.0) on six of seven dimensions—Conciseness being the sole exception (3.13 ± 0.74). Counterfactual Reasoning received the lowest mean (1.77 ± 1.34), and Evidence Linkage (2.47 ± 1.25) and Overall Clinical Utility (2.07 ± 1.08) were also rated below the midpoint, indicating that the AI-generated explanations fell below the physicians’ standard of adequacy across multiple dimensions. Mean ratings were broadly similar across reviewers, although one reviewer applied a more stringent scoring pattern for Clinical Specificity and Reasoning Coherence (Table 5).

**Figure 5:**
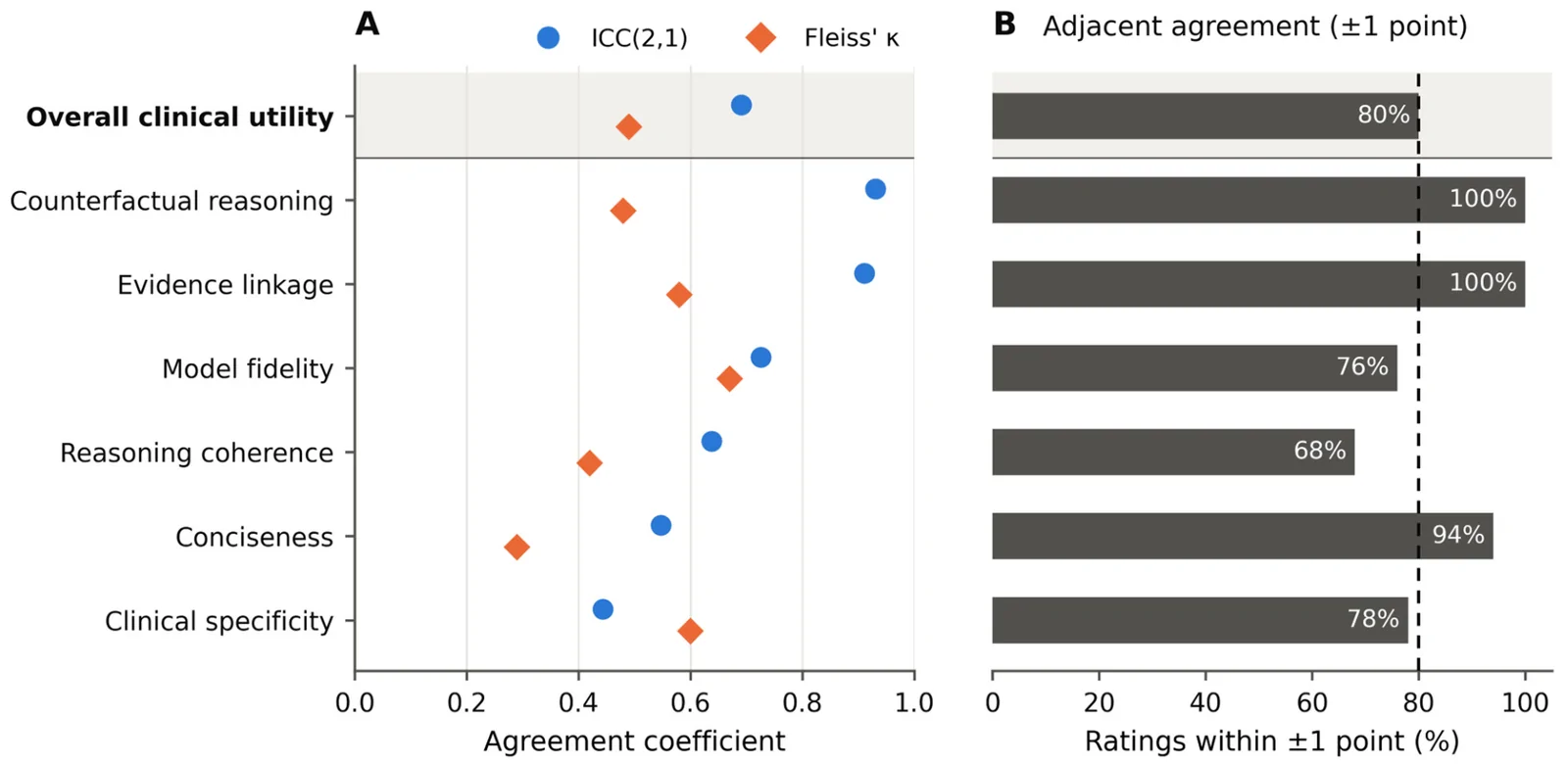
Inter-rater reliability across the seven evaluation dimensions (50 cases, three physician raters, ratings on a 1–5 scale). Overall clinical utility is highlighted; the other dimensions are sorted by ICC. (**A**) ICC(2,1) and Fleiss’ κ. (**B**) Percentage of cases in which all three ratings fell within ±1 point; the dashed line marks 80%.

The highest agreement was observed for Counterfactual Reasoning (ICC(2,1) = 0.931) and Evidence Linkage (ICC(2,1) = 0.911), indicating excellent consistency among reviewers (Figure 5). Model Fidelity (ICC = 0.726), Overall Clinical Utility (ICC = 0.691), Reasoning Coherence (ICC = 0.638), and Conciseness (ICC = 0.547) showed moderate agreement. Clinical Specificity exhibited the lowest reliability (ICC = 0.443), attributable to range restriction in one rater who exclusively used scores of 1 or 2 for that dimension, a calibration difference rather than a conceptual disagreement, as pairwise weighted κ_w_ between the two unrestricted raters was 0.647. Fleiss’ κ values ranged from 0.29 to 0.67, indicating fair-to-substantial categorical agreement across dimensions.

A complementary adjacent-agreement analysis demonstrated that reviewer disagreement was generally small in magnitude. All three reviewers scored within ±1 point of each other in 100% of cases for Evidence Linkage and Counterfactual Reasoning, 94% of cases for Conciseness, and 80% of cases for Overall Clinical Utility. Agreement was lowest for Reasoning Coherence (68%) and Clinical Specificity (78%), consistent with the lower ICC estimates observed for these dimensions.

**Figure 6:**
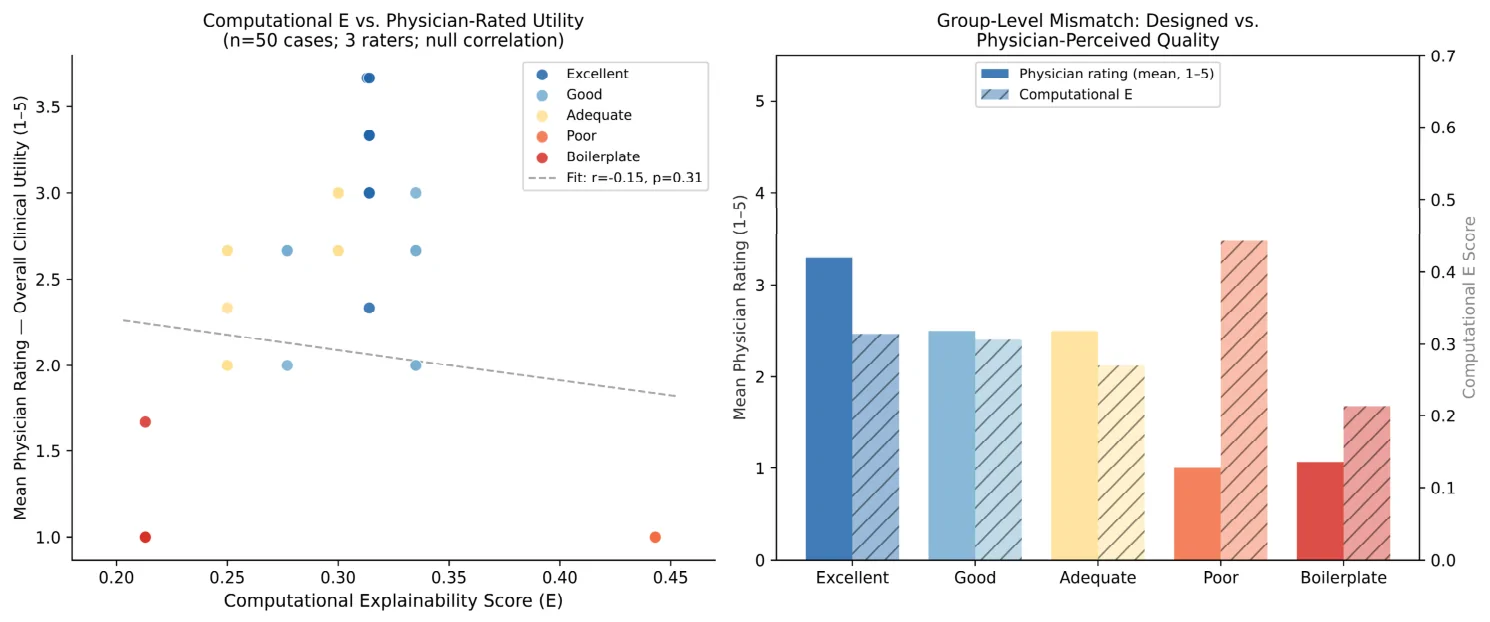
Comparison of computational explainability scores and physician–rated clinical utility.

To evaluate convergent validity, physician-rated Overall Clinical Utility was compared with the computational explainability score (*E*). No statistically significant association was observed between computational *E* and mean physician utility ratings (Pearson r = −0.15, *p* = 0.31; Spearman ρ = 0.06, *p* = 0.67; Figure 6), consistently across all three raters (r: −0.12 to −0.16, all *p* > 0.25) and all seven dimensions (all |r| < 0.20, all *p* > 0.18). The selected cases also did not reproduce their target *E* levels: cases selected as “Poor” (target *E* ≈ 0.25) had the highest observed composite *E* (0.443) and the lowest physician utility (Ū = 1.00/5), because the TF-IDF evidence sub-component rewards verbatim mention of features regardless of clinical correctness; “Excellent” cases (target ≈ 0.80) had observed *E* 0.314 and the highest utility (Ū = 3.30/5); and “Boilerplate” cases (target ≈ 0.12) had observed *E* 0.213 and Ū = 1.07/5. The computational metric therefore did not capture the aspects of explanation quality that these physicians valued.

Safety concern ratings showed moderate agreement among reviewers. Overall, 51.3% of case-rater evaluations were flagged as potential safety concerns, and 56% of cases were identified as safety concerns by majority vote. Three-way unanimous agreement was observed in 58% of cases. Pairwise agreement ranged from substantial (κ = 0.647 between two reviewers) to fair (κ = 0.283–0.396 for comparisons involving the third reviewer), indicating variability in reviewer thresholds for identifying safety risks.

These results must be read in light of the study design (Section 2.12). The rated explanations came from a MIMIC-III mortality model whose attribution was uncentered and whose text contained factual errors, and raters knew each outcome and whether the model was correct. Low utility ratings may therefore reflect a defective explanation generator as much as a defective *E* metric. The study shows that physicians rated these explanations as poor and that *E* did not track their ratings; it does not show how *E* would perform on correct explanations.

Cases flagged as safety concerns did not receive statistically significantly lower Overall Clinical Utility ratings than non-flagged cases (mean 2.01 vs. 2.15; Mann–Whitney U = 282, *p* = 0.61, two-sided). This suggests that perceived clinical utility and safety concerns represent distinct constructs: explanations may be considered clinically unhelpful without necessarily being viewed as unsafe, and vice versa.

### 3.16. Risk-Controlled Deployment

On the primary cohort (prolonged ICU stay; model error rate 22.2%), the default trust gate deployed 76.5% of predictions with an error rate of 18.4% among them, but with no guarantee on that error rate (Table 6). With a guaranteed error budget, a threshold could be certified at α = 0.10 in only 1 of 1000 splits; on average, 0.7% of predictions were deployed at α = 0.15 and 15.0% at α = 0.20, with no certifiable threshold in 49.9% of splits at α = 0.20; the error rate among deployed predictions was 13.3% at α = 0.20. Selective risk exceeded α in at most 0.8% of splits. Across the 18 scenario-model combinations, the mortality models were certified at α = 0.10 while deploying 86–97% of predictions, whereas the prolonged-stay models deployed 0–1% at α = 0.10 and 0–68% at α = 0.20; the eICU prolonged-stay MLP could not be certified at any budget. Selective risk exceeded α in at most 1.5% of splits in any combination (Supplementary File S1, Section S14). The certified threshold therefore distinguishes models that can operate autonomously at a stated error budget from models that cannot, which the default trust gate does not.

## 4. Discussion

We formalized a deployment rule in which the trust score required for autonomous use depends on system transparency and evaluated it retrospectively. The central finding is negative and specific: in the present implementation, the rule’s per-case decisions are those of a confidence threshold. *E* and *S* vary too little between patients to change which predictions are deferred; they shift the confidence cut-off between models, and re-optimizing the weights on outcome data assigns all weight to confidence. The framework is a governance and decision-support mechanism whose behavior has been examined only retrospectively; it is not an established safety mechanism, and prospective validation with patient-centered outcomes is required before deployment decisions rely on it.

What the framework does provide is an explicit, auditable policy for choosing the operating point of a selective predictor. The threshold τ(*E*) converts a model-level transparency assessment into a confidence cut-off, and thus into a coverage level, through parameters (θ_0_, κ, weights) that a governance committee can set, document, and revise. κ controls pass rates continuously (Figure 2 and Figure 3), up to a ceiling beyond which no prediction can pass (Section 3.11). This makes a previously implicit institutional choice explicit. Whether tying coverage to transparency is desirable depends on whether *E* measures something clinicians value, which our data do not show.

Our results suggest a division of labor in governance. At the system level, transparency review, validation, and audit can inform whether a model may operate autonomously at all and under what error budget—for example, a stricter α for systems that have not passed an institution’s transparency review. This is a policy choice, not an estimated quantity, and the computational *E* evaluated here is not suitable for it. At the case level, deployment should rest on calibrated confidence with a certified threshold (Section 3.16), because confidence is what carries information about errors. In this design, transparency acts on the error budget rather than on the per-case threshold, and the certified rule shows when a model—such as the prolonged-stay model here—cannot support autonomous operation at an acceptable error rate.

The linear, multiplicative, and outcome-fitted interaction formulations ranked predictions identically (Table 3), so the choice among them matters only for where a fixed threshold falls. The linear form is preferable for governance because its threshold can be read directly as a confidence cut-off.

The underlying models transferred with modest loss of discrimination between MIMIC-III and MIMIC-IV (AUC difference 0.011) and between MIMIC-IV and eICU (mean cross-institutional AUC 0.816 vs. 0.836 internal), although the eICU prolonged-stay MLP performed poorly (AUC 0.55). Default pass rates depended on the endpoint’s prevalence and each model’s confidence distribution (37–87% for prolonged stay; 70–98% for mortality; Table 2), so governance parameters must be set locally rather than transferred.

A central practical consideration is the trade-off between autonomous deployment and clinician review workload. At default thresholds, 23.5% of primary-cohort prolonged-stay predictions were deferred with per-patient scoring (Table 1) and 40–63% in the MIMIC-IV prolonged-stay scenarios (Table 2); 11–25% of MIMIC-IV mortality predictions were deferred. Because the deferred set is the least-confident set, any institution adopting the rule should compare it directly with a calibrated confidence threshold at the same workload; a pragmatic pathway is local retrospective evaluation, silent deployment with human override, and prospective audit of outcomes in deferred and deployed predictions.

Three physicians rated AI-generated explanations on seven predefined dimensions. Agreement was excellent for Evidence Linkage and Counterfactual Reasoning (ICC 0.91–0.93), moderate for Model Fidelity, Overall Clinical Utility, Reasoning Coherence, and Conciseness (ICC 0.55–0.73), and lowest for Clinical Specificity (ICC 0.44).

Computational *E* was not correlated with physician-rated utility (r = −0.15, *p* = 0.31), and cases selected for low *E* scored higher on *E* than cases selected for high *E*. Because the rated explanations contained generator errors and raters were not blinded (Section 2.12), this result cannot separate a defective metric from defective explanations; both need to be fixed. Its practical implication is nonetheless clear: the current *E* should not be used to set deployment thresholds. The next step is to regenerate explanations from the primary model with centered attribution, verify them clinically, and repeat the rating blinded to outcomes and model correctness before *E* is refined against those ratings.

Relative to existing frameworks, our contribution is narrower than a new gating mechanism. Algebraically, the rule is a fixed threshold on a linear score in which *E* carries weight β + κ (Section 2.4), and with near-constant *E* and *S,* it reduces, within a model, to selective prediction on confidence [27]. What the framework adds is a documented, tunable link between a model-level transparency assessment and the coverage at which the model operates, expressed through parameters that institutions can audit, together with a deployment threshold whose error rate is certified on data (Table 7). Existing governance frameworks generally state principles or report confidence and explainability separately [5]; our formulation makes the trade-off explicit but does not, on current evidence, improve which predictions are deferred.

Our results place the framework within the literature on selective prediction and deferral. Complementarity-driven deferral to clinicians improves diagnostic reliability in AI-enabled chest radiograph interpretation [28], and selective classification supplies the machine-learning foundation for declining to act on uncertain predictions [27]; per case, our rule is an instance of the latter, and the certified threshold of Section 2.13 applies its guaranteed-risk form. Conformal prediction offers related distribution-free guarantees [29], and the DECIDE-AI guideline defines the staged evaluation that prospective use should follow [30]. A transparency component could add value only if it carries per-case information about error that confidence does not; testing that requires a validated, patient-level *E*.

The proposed framework also aligns conceptually with emerging regulatory approaches for trustworthy AI [31,32]. By linking deployment authority to both predictive confidence and transparency, the adaptive threshold supports risk-based governance strategies consistent with evolving regulatory guidance for clinical AI, including FDA Software as a Medical Device (SaMD) principles and the European Union AI Act. Importantly, the threshold values presented here are illustrative rather than prescriptive; the framework is intended to provide a mechanism through which institutions can calibrate, document, and justify deployment policies according to their local clinical environment, operational requirements, and risk tolerance rather than relying on universal thresholds.

From an implementation perspective, adoption would require local retrospective validation, local setting of error budgets and governance parameters, and institution-specific evaluation of explanation quality before integration into clinical workflows [18]. The framework is therefore better framed as a general methodology for governance and should not be viewed as a fixed set of deployment parameters.

Several limitations warrant consideration. First, all data were retrospective US ICU data, and the default weights (α = 0.60, β = 0.24, γ = 0.16) are policy choices that re-optimization did not support (Section 3.3); any adopter must set and justify weights and thresholds locally. Second, the certified threshold is conservative: its guarantee requires exchangeability between calibration and deployment patients, and the calibration sample (500 patients per split) and the correction for testing 26 thresholds limit the coverage that can be certified; larger local calibration samples would certify more.

Third, although physician reviewers demonstrated moderate to excellent inter-rater reliability across most evaluation dimensions, the computational explainability metric showed poor convergent validity with physician-rated Overall Clinical Utility, indicating that the current implementation does not capture the aspects of explanation quality most relevant to clinical decision-making. The explainability component relies primarily on computational proxies, including TF-IDF similarity, keyword matching, and rule-based heuristics [33], which are objective and reproducible but cannot represent the contextual reasoning, actionability, and clinical relevance that physicians consider when evaluating AI-generated explanations. The clinician study included only three physician reviewers and 50 clinical cases; raters saw each case’s outcome and whether the model’s prediction was correct, two calibration examples reappeared among the evaluated cases, and physicians could not fully assess model fidelity without access to the model. A blinded, outcome-masked study without calibration-case overlap, larger and multicenter, is needed to characterize physician agreement and to develop clinician-informed calibration of the explainability metric.

In addition, the semantic similarity component currently relies on simplified guideline representations and keyword-based matching rather than structured medical ontologies or knowledge graphs, such as SNOMED CT or UMLS [34]. While this implementation demonstrates the operational feasibility of incorporating guideline alignment into the trust framework, more comprehensive ontology-driven approaches and dynamically updated clinical knowledge sources will likely improve robustness and generalizability.

In addition, *E* takes few discrete values determined by the explanation template and can shift by 0.10 with minor pipeline changes (Section 3.2). Rebuilding *S* from authoritative guideline sources, regenerating explanations with centered attribution, a blinded and outcome-masked clinician study, testing whether a patient-level *E* adds information about error beyond confidence, and numerical instantiation of the PAC-Bayesian bound remain future work.

Finally, the present framework was developed and evaluated using English-language ICU data from a limited number of prediction tasks. Validation across additional clinical specialties, healthcare systems, languages, and real-world deployment settings will be necessary to establish the framework’s broader applicability and operational impact.

## 5. Conclusions

We formalized and retrospectively evaluated a rule that links the deployment authority of a clinical AI system to its transparency, using 15,000 ICU patients from MIMIC-IV, MIMIC-III, and eICU and three model architectures. The rule gives institutions an explicit, tunable, and auditable way to set how many predictions are deployed without review. In its current implementation, however, its per-case decisions are identical to those of a calibrated confidence threshold: the explainability and guideline-alignment components vary too little between patients to change which predictions are deferred, re-optimized weights favor confidence alone, and computational explainability did not track physician ratings of explanations that themselves contained errors. Before transparency can justify a lower deployment threshold, explanation quality must be measured per patient, validated against blinded clinician judgment, and shown to identify errors that confidence misses. Until then, transparency is better used at the system level to set the error budget under which a model may operate, while per-case deployment rests on calibrated confidence with a certified threshold; in our data, such a threshold certified the mortality models at a 10% error budget while deploying 86–97% of predictions but deployed on average only 15% of primary prolonged-stay predictions at a 20% budget.

## Figures and Tables

**Table 3:** Linear, multiplicative, and outcome-fitted interaction formulations on the primary held-out cohort (predicted-class confidence definition; per-patient *E* and *S*; *n* = 1000). Agreement and Cohen’s κ compare deploy/defer decisions with the linear gate at matched coverage (765 deployed).

Score	AURC	Agreement with Linear	Cohen’s κ	r with Linear	Errors Deployed
Linear T (proposed)	0.141	—	—	—	141
Multiplicative	0.142	100.0%	1.00	0.999	141
Interaction (fitted to outcomes)	0.141	100.0%	1.00	0.987	141
Confidence C only	0.140	99.4%	0.98	0.999	140

AURC, area under the risk–coverage curve; r, Pearson correlation of scores. Interaction model coefficients (logistic regression on unscaled inputs): *C* 3.21, *E* 0.60, *S* −0.35, *C* × *E* 1.84, *C* × *S* 2.11, *E* × *S* 0.39.

**Table 4:** S-component ablation under the predicted-class confidence definition: full score versus C + E only with renormalized weights. Primary cohort with per-patient E and S (*n* = 1000) and the 18 scenario-model combinations of Table 2 (model-level E and S).

Setting	Model	Pass %, Full	Pass %, No-S	Agreement	Cohen’s κ	Decisions Changed
Primary cohort (per-patient E, S)	LR	76.5	73.4	96.9%	0.92	31
MIMIC-IV internal (mortality)	LR	85.4	83.0	97.6%	0.91	36
	GB	89.1	89.1	100%	1.00	0
	MLP	75.4	75.4	100%	1.00	0
MIMIC-IV internal (prolonged)	LR	37.4	37.4	100%	1.00	0
	GB	39.1	39.1	100%	1.00	0
	MLP	60.1	60.1	100%	1.00	0
eICU internal (mortality)	LR	88.1	88.1	100%	1.00	0
	GB	80.5	80.5	100%	1.00	0
	MLP	88.8	88.8	100%	1.00	0
eICU internal (prolonged)	LR	46.1	46.1	100%	1.00	0
	GB	48.7	48.7	100%	1.00	0
	MLP	86.5	86.5	100%	1.00	0
MIMIC-IV → eICU (mortality)	LR	90.6	90.6	100%	1.00	0
	GB	80.4	80.4	100%	1.00	0
	MLP	97.6	96.8	99.2%	0.85	41
eICU → MIMIC-IV (mortality)	LR	80.0	80.0	100%	1.00	0
	GB	70.4	70.4	100%	1.00	0
	MLP	79.3	79.3	>99.9%	1.00	1

LR, logistic regression; GB, gradient boosting; MLP, multilayer perceptron; no-S, trust score without the semantic similarity component, with renormalized weights; Agreement, percentage of identical deploy/defer decisions.

**Table 5:** Physician evaluation across seven dimensions.

Dimensions	Mean (SD)	Median [Range]	ICC(2,1)	Fleiss’ κ
Evidence Linkage (1–5)	2.47 (1.25)	3 [1–4]	0.911	0.58 (Moderate)
Reasoning Coherence (1–5)	2.21 (1.28)	2 [1–5]	0.638	0.42 (Moderate)
Clinical Specificity (1–5)	1.93 (1.13)	2 [1–5]	0.443	0.60 (Substantial)
Conciseness (1–5)	3.13 (0.74)	3 [1–4]	0.547	0.29 (Fair)
Counterfactual Reasoning (1–5)	1.77 (1.34)	1 [1–5]	0.931	0.48 (Moderate)
Model Fidelity (1–5)	2.46 (1.35)	3 [1–5]	0.726	0.67 (Substantial)
Overall Clinical Utility (1–5)	2.07 (1.08)	2 [1–4]	0.691	0.49 (Moderate)

SD, standard deviation; range, minimum–maximum; ICC(2,1), two-way random-effects intraclass correlation coefficient for single measurements (absolute agreement); κ, Fleiss’ kappa coefficient.

**Table 6:** Default trust gate and risk-controlled deployment on the primary held-out cohort (MIMIC-IV, prolonged ICU stay, *n* = 1000; 1000 random splits into calibration and evaluation halves of 500 each; δ = 0.05). Values are means across splits with 2.5th–97.5th percentiles in parentheses, computed on the evaluation halves; selective risk is the error rate among deployed predictions, summarized over splits with at least one deployed prediction.

Rule	Error Budget α	Predictions Deployed	Selective Risk	Splits with No Deployment	Splits with Selective Risk > α
Default trust gate (θ_0_ = 0.5, κ = 0.20)	—	76.5% (74.2–79.2)	18.4% (15.7–21.0)	0%	—
Certified threshold	0.10	0.0% (0.0–0.0)	— (deployed in 1 split)	99.9%	0.1%
Certified threshold	0.15	0.7% (0.0–10.2)	12.1% (9.1–18.5)	94.6%	0.8%
Certified threshold	0.20	15.0% (0.0–66.2)	13.3% (6.5–19.4)	49.9%	0.5%

**Table 7:** Comparison with existing trust framework categories in healthcare AI.

Dimension	Static Threshold	Confidence-Only	Explainability-Only	Qualitative Ethics	Ours
**Formal specification**	Fixed cut-off	AUC/ECE	Feature importance	Principles only	Linear score and threshold (equivalent to a confidence cut-off *c*_min_(E, S)); certified threshold with a finite-sample error guarantee
**Adaptive threshold**	No	No	No	No	τ(E), set at model level; error budget α set by policy
**Components**	C only	C only	E only	None	C + E + S (E and S not yet validated; weights fixed by policy)
**Cross-dataset evaluation**	No	No	No	No	MIMIC-III, MIMIC-IV, eICU (model discrimination)
**Deployment governance**	Post hoc	Post hoc	Post hoc	Principles only	System-level review sets α; case-level certified confidence threshold

AUC, area under the receiver operating characteristic curve; *C*, confidence; *E*, explainability; ECE, expected calibration error; *S*, semantic similarity; τ(*E*), adaptive trust threshold; *c*_min_, implied confidence cut-off.

## Data Availability

MIMIC-III, MIMIC-IV, and eICU data are available through PhysioNet (https://physionet.org/content/mimiciii/ (accessed on 9 October 2026), https://physionet.org/content/mimiciv/ (accessed on 9 October 2026), and https://physionet.org/content/eicu-crd/ (accessed on 9 October 2026)) following completion of required training and data use agreements. This study used MIMIC-IV version 3.1 (https://doi.org/10.13026/kpb9-mt58). Framework implementation code, including all computational methods for trust score calculation, explainability approximation, and semantic similarity measurement, and synthetic validation examples are available on GitHub (https://github.com/yuyunguo/adaptive-trust-threshold, accessed on 9 October 2026).

## Supplementary Materials

The following supporting information can be downloaded at: https://www.mdpi.com/article/10.3390/healthcare14213736/s1, Supplementary File S1: Supplementary methods and analyses; Supplementary File S2: Clinician evaluation workbook; TRIPOD + AI checklist.
