# Supplementary material for: Governing Clinical AI Deployment: A Mathematical Framework for Transparency-Dependent Autonomy
Source: Healthcare (Basel). 2026 Nov 3;14(21):3736. doi: 10.3390/healthcare14213736 (PMC13762715; doi:10.3390/healthcare14213736)
Supplement: Supplementary file 1 [file healthcare-14-03736-s001.zip › Supplementary_File_S1.pdf]

Supplementary Materials: Governing Clinical AI Deployment: A Mathematical Framework for Transparency-Dependent Autonomy

Yunguo Yu, MD, PhD[1], Bernardo Gabriele Collaco, MD[2], Srinivasagam Prabha, PhD[2], Syed Ali Haider, MBBS[2], Carina Rosa Malena, MD[2], Yogesh Parte, PhD[1], Nadia G. Wood, MS[3], Bradley C. Leibovich, MD[5], and Antonio Jorge Forte, MD, PhD*[1,2,4]

[1]Center for Digital Health, Mayo Clinic, Rochester, MN 55905, USA
[2]Division of Plastic Surgery, Mayo Clinic, 4500 San Pablo Rd S, Jacksonville, FL 32224, USA
[3]Department of Radiology AI IT, Mayo Clinic, Rochester, MN 55905, USA
[4]Department of Artificial Intelligence and Informatics, Mayo Clinic, Jacksonville, Florida, USA
[5]Department of Urology, Mayo Clinic, Rochester, MN 55905, USA

Contents

S1 Weight Sensitivity Analysis **4**
S1.1 Configuration Grid . . . 4
S1.2 Optimization Metrics . . . 4
S1.3 Optimal Configuration Results . . . 4
S1.4 Sensitivity Summary . . . 5
S1.5 Threshold Parameter Sensitivity (κ, θ_0) . . . 5

S2 Trust Convergence Validation **6**
S2.1 Validation Results . . . 6

S3 Framework Architecture **6**

S4 Clinical Data Validation **7**
S4.1 Explainability Metric Development and Validation . . . 7
S4.2 Computational Validation Approach . . . 7

*Corresponding author. Division of Plastic Surgery, Mayo Clinic, 4500 San Pablo Rd S, Jacksonville, FL 32224, USA. Email: ajvforte@yahoo.com.br. ORCID: https://orcid.org/0000-0003-2004-7538

S4.3 Anti-Gaming Validation . . . 8
S4.4 Limitations Acknowledged . . . 8

S5 Statistical Analysis **8**
S5.1 Database . . . 8
S5.2 Patient Cohort . . . 8
S5.3 Feature Engineering . . . 9
S5.4 Data Leakage Prevention . . . 10
S5.5 Primary Analysis . . . 10

S6 Reproducibility Protocol **10**
S6.1 Multiple Independent Executions . . . 10
S6.2 Statistical Analysis . . . 10
S6.3 Ethics and Data Use . . . 10
S6.4 Code Availability . . . 11

S7 Detailed Component Definitions and Mathematical Formulations **11**
S7.1 Confidence Component (C) — Detailed Specification . . . 11
S7.1.1 Current Implementation . . . 11
S7.1.2 Model Training Details . . . 11
S7.1.3 Model Performance . . . 12
S7.2 Explainability Component (E) — Detailed Algorithm . . . 13
S7.2.1 Complete 6-Component Algorithm . . . 13
S7.2.2 Component Definitions . . . 13
S7.2.3 Provenance of Supplementary File S2 . . . 14
S7.3 Semantic Similarity Component (S) — Detailed Specification . . . 14
S7.3.1 Enhanced Semantic Similarity Calculation . . . 14
S7.3.2 Knowledge Sources . . . 14
S7.3.3 Component Definitions . . . 15
S7.4 Mathematical Properties and Theoretical Guarantees . . . 15
S7.5 Adaptive Trust Threshold Integration . . . 16

S8 Trust Framework Implementation **16**
S8.1 SimplifiedTrustAnalyzer . . . 16
S8.2 ClinicalExplainabilityDashboard . . . 17
S8.3 ClinicalTrustMetrics . . . 17

S9 Mathematical Proofs and Convergence Theory **17**
S9.1 Theorem 1: Trust Calibration Convergence (Robbins-Monro) . . . 17
S9.1.1 Required Assumptions . . . 17
S9.1.2 Detailed Proof . . . 17
S9.2 Theorem 2: Trust Score Monotonicity . . . 18
S9.2.1 Required Assumptions . . . 18
S9.2.2 Detailed Proof . . . 18
S9.3 Theorem 3: Clinical Safety Bounds (PAC-Bayes) . . . 19
S9.3.1 Required Assumptions . . . 19
S9.3.2 Detailed Proof . . . 19

S10 Deployment Simulation Methodology **20**

S10.1 Simulation Design 20
S10.2 Assumptions and Rationale 20
S10.3 Calculation 21
S10.4 Sensitivity to Human Review Accuracy 21
S10.5 Limitations of the Simulation 21

S11 Failure Mode Analysis: Clinical Case Studies **22**

S12 Preliminary Cross-Cohort Analysis within MIMIC-III **22**
S12.1 Within-Cohort Performance 22
S12.2 Key Findings 22
S12.3 Limitations 23

S13 Secondary Endpoint: Mortality **23**
S13.1 Results 23
S13.2 Comparison 23
S13.3 Data and Validation Limitations 24
S13.4 Component Metric Limitations 24
S13.5 BERTScore Evidence Linkage Validation 25
S13.6 Cross-Dataset Validation: MIMIC-III vs. MIMIC-IV 26
S13.7 External Cross-Institutional Validation: MIMIC-IV vs. eICU 27
S13.8 Threshold Discriminability: Full Sensitivity Analysis 28
S13.9 Linear vs. Nonlinear Trust Formulation: Full Results 28
S13.10 S-Component Ablation: Full Results 29
S13.11 Framework Assumptions 29
S13.12 Deployment Readiness 29
S13.13 Generalizability Constraints 30
S13.14 Theoretical Limitations 30
S13.15 Future Work 30

S14 Additional Analyses and Results under the Positive-Class Confidence Definition **31**
S14.1 Primary-Cohort Features 31
S14.2 Outcome-Based Weight Re-optimization 32
S14.3 Same-Threshold Multiplicative Agreement and κ Sweep (18 Combinations) 32
S14.4 Risk-Controlled Deployment: All Scenarios 33
S14.5 Validation Scenarios under the Positive-Class Confidence Definition 34

S1 Weight Sensitivity Analysis

Note on the weight search. The grid search described in this section was run without outcome data: the false-accept and false-reject terms were fixed formulas with added random noise (FAR $= 0.15(1-0.6)+\mathcal{N}(0,0.02)$, FRR $= 0.1\times 0.6+\mathcal{N}(0,0.02)$), the calibration term was a histogram-uniformity heuristic with added noise, the MAE term was computed against $\tau(E)$ rather than against prediction correctness, FAR and FRR used a fixed threshold of 0.6 rather than $\tau(E)$, and C was the positive-class probability. The weights $(0.60, 0.24, 0.16)$, and the false-accept (4.2%) and false-reject (7.7%) rates produced by this procedure, are therefore not estimates from outcomes. The main text uses these weights as fixed governance parameters; an outcome-based weight search is reported in Section S14.

We conducted comprehensive sensitivity analysis across 162 weight configurations using systematic grid search **for this specific dataset and clinical context** (MIMIC-III intensive care unit (ICU) mortality prediction with logistic regression architecture).

S1.1 Configuration Grid

- α (confidence weight): $[0.2, 0.6]$ with step size 0.029
- β (explainability weight): $[0.1, 0.5]$ with step size 0.029
- γ (semantic similarity weight): derived as $\gamma = 1-\alpha-\beta$, subject to $\gamma \geq 0.1$
- **Valid configurations:** 162 feasible (α, β) pairs after enforcing $\gamma \geq 0.1$ and both bounds

All 162 configurations satisfy $\alpha+\beta+\gamma = 1.0$ with $\alpha, \beta, \gamma \geq 0$. The grid does not include all Cartesian combinations of the two ranges because $\gamma < 0.1$ combinations are excluded as they would assign insufficient weight to the semantic alignment component.

S1.2 Optimization Metrics

- Mean trust score performance
- Calibration error minimization
- False-accept rate (target: $< 2\%$)
- False-reject rate (target: $< 5\%$)

S1.3 Optimal Configuration Results

- **Optimal Weights:** $\alpha = 0.60$, $\beta = 0.24$, $\gamma = 0.16$ (**dataset and context-dependent**)
- **Performance Metrics (from optimal configuration evaluation):**
  - Mean Trust Score: 0.523 [Note: This value is from the weight sensitivity analysis optimal configuration evaluation. The final validation result across 5,000 patients is 0.239 (standard deviation [SD]: 0.098) for the secondary mortality endpoint, and 0.364 (SD: 0.145) for the primary prolonged ICU stay endpoint.]

- Trust-Acceptance MAE: 0.338
- False-Accept Rate: 4.2%
- False-Reject Rate: 7.7%

S1.4 Sensitivity Summary

- **Trust Score Variance**: 0.0042 across all configurations
- **Most Sensitive Component**: β (explainability) with variance 0.013
- **Least Sensitive Component**: γ (semantic similarity) with variance 0.011

Important Note on Dataset Dependency: These optimal weights are specific to the MIMIC-III ICU mortality prediction context and the logistic regression model architecture employed. Different clinical domains, patient populations, or AI architectures may require re-optimization through domain-specific sensitivity analysis. The framework provides a **robust methodology** for trust validation rather than fixed parameters.

S1.5 Threshold Parameter Sensitivity (κ, θ_0)

The adaptive threshold $\tau(E) = \theta_0 + \kappa(1 - E)$ has two parameters: the baseline threshold θ_0 (minimum trust at maximum explainability) and the transparency penalty κ. Both are institutional policy parameters rather than model-derived constants. To characterize their effect on deployment decisions, we conducted a 2D sensitivity sweep across $\kappa \in [0, 0.50]$ and $\theta_0 \in [0.3, 0.7]$, computing the pass rate ($T \geq \tau(E)$) for each combination.

Table S1: Pass rate as a function of κ (with $\theta_0 = 0.5$). Qualitative descriptors refer to the prolonged stay endpoint (32% prevalence) and use the positive-class confidence definition ($C = \hat{p}$); pass rates under the predicted-class definition ($C = \max(\hat{p}, 1 - \hat{p})$) for all scenarios appear in main-text Table 2. Quantitative sweep results across all 12 scenario-model combinations are provided in Table S6.

κ	τ Range	Pass Rate (prolonged stay)	Assessment
0.00	$[0.50, 0.50]$	near-100%	No transparency incentive
0.10	$[0.50, 0.60]$	moderate-high	Weak transparency incentive
**0.20**	$[0.50, 0.70]$	**variable by endpoint**	**Recommended default**
0.30	$[0.50, 0.80]$	low	Strong penalty for opaque systems
0.40	$[0.50, 0.90]$	near-0%	Prohibitive for most opaque systems

At the default configuration ($\theta_0 = 0.5$, $\kappa = 0.20$), the threshold ranges from $\tau = 0.50$ (system with $E = 1.0$) to $\tau = 0.70$ (system with $E = 0.0$), representing a 20% confidence penalty for fully opaque systems. The 2D sensitivity surface (main-text Figure 2) shows that θ_0 controls the overall deployment stringency, while κ controls the transparency gradient: higher κ amplifies the disadvantage of opaque systems. Institutions can calibrate these parameters to local risk preferences: a conservative institution might increase both θ_0 and κ for high-stakes settings, while a permissive institution might lower them for low-risk applications.

The statistical profile of flagged versus non-flagged cases at the default configuration confirms discriminability. For the prolonged stay endpoint: flagged cases have significantly lower confidence (C: 0.32 vs. 0.76, $p < 0.001$), lower explainability (E: 0.43 vs.

0.63, $p < 0.001$), and lower trust scores (T: 0.40 vs. 0.71, $p < 0.001$). Semantic similarity (S) showed no significant difference ($p = 0.06$), as expected given its narrow distribution. For the cross-dataset mortality scenarios (12 scenario-model combinations), flagged vs. passed differences were: C (0.06–0.09 vs. 0.35–0.65), E (0.25–0.38 vs. 0.38–0.42), T (0.20–0.25 vs. 0.55–0.72), all $p < 0.001$. The trust margin $T - \tau(E)$ provided the strongest separation ($p < 10^{-50}$), confirming that the adaptive threshold identifies cases with simultaneously low confidence and low transparency.

S2 Trust Convergence Validation

Convergence validation requires simultaneous satisfaction of clinical thresholds:

$$\text{Convergence\_Achieved} = (\text{Trust\_Framework\_Integrated}) \wedge (\text{Mathematical\_Convergence}) \wedge (\text{Criteria\_Met}) \quad \text{(S1)}$$

S2.1 Validation Results

- Trust Framework Integrated: True (100% of validation runs)
- Mean Trust Score: 0.364 (SD: 0.145) for the primary prolonged ICU stay endpoint (secondary mortality endpoint: 0.239, SD: 0.098) — both under the positive-class confidence definition ($C = \hat{p}$); see main-text Table 2 for the predicted-class definition
- Mathematical Convergence: True (convergence criteria met)
- Convergence criteria were met in all independent validation runs

S3 Framework Architecture

Our trust framework implements a modular architecture with specialized components:

1. **Data Processing Module:** Demographic and administrative data extraction from the MIMIC-III PostgreSQL database with comprehensive quality validation.
2. **Clinical Prediction Module:** In-hospital mortality risk stratification with explainable AI capabilities. The module achieved an area under the receiver operating characteristic curve (AUC-ROC) of 0.85 (95% confidence interval [CI]: 0.82–0.88) with calibration (expected calibration error [ECE]: 0.044) across the validation cohort.
3. **Trust Analysis Engine:** Trust score computation through three specialized components:
    - **SimplifiedTrustAnalyzer:** Core trust computation engine with multi-dimensional trust assessment
    - **ClinicalExplainabilityDashboard:** Explainability scoring through feature usage analysis

- **ClinicalTrustMetrics:** Trust metadata framework with clinical threshold validation

4. **Adaptive Threshold Controller:** Trust-based decision support implementing the adaptive threshold $\tau(E) = 0.5 + 0.2 \times (1 - E)$ to determine deployment eligibility based on transparency-dependent autonomy.

Note: While the framework is designed to support multi-system orchestration, the current validation focuses on single-model predictions. Future work will validate the framework's extension to heterogeneous AI system orchestration with conflict resolution and communication protocols.

S4 Clinical Data Validation

S4.1 Explainability Metric Development and Validation

The 6-component metric was designed to capture key dimensions of clinical explanation quality, with component weights selected to balance the relative importance of each dimension:

- Evidence linkage (25%): Highest weight reflecting the importance of grounding explanations in clinical data
- Reasoning coherence (20%) and clinical specificity (20%): Equal weights for logical flow and clinical terminology
- Conciseness (15%): Moderate weight for optimal information density
- Counterfactual reasoning (10%) and model fidelity (10%): Lower but important weights for uncertainty acknowledgment and model alignment

S4.2 Computational Validation Approach

Each component uses established, reproducible computational methods:

- **Evidence Linkage (25%)**: term frequency-inverse document frequency (TF-IDF) cosine similarity between explanation text and clinical features (threshold: 0.3)
- **Reasoning Coherence (20%)**: Causal term frequency analysis using validated medical reasoning vocabulary
- **Clinical Specificity (20%)**: Medical terminology validation using keyword matching against hardcoded clinical feature categories. **Note**: Current implementation uses hardcoded dictionaries rather than Systematized Nomenclature of Medicine—Clinical Terms (SNOMED CT) integration; future work will incorporate SNOMED CT and Unified Medical Language System (UMLS) for comprehensive terminology validation.
- **Conciseness (15%)**: Length optimization with penalty parameter ($\lambda = 0.05$) based on cognitive load literature (optimal: 30 words)

- **Counterfactual Reasoning (10%)**: Differential diagnosis pattern detection and confidence interval analysis
- **Model Fidelity (10%)**: Perturbation-based surrogate model agreement (30 perturbations, $\alpha = 1.0$)

S4.3 Anti-Gaming Validation

Computational robustness testing demonstrated:

- Irrelevant feature addition decreased E by 12%
- Duplicate term repetition showed no score inflation (±0.02 range)
- Boilerplate text copying decreased E by 8%
- Random numeric injection had minimal impact (±0.03 range)

S4.4 Limitations Acknowledged

- Computational validation alone cannot provide inter-rater reliability statistics (intraclass correlation coefficient [ICC], Cronbach's α) or empirical clinician-correlation data; the physician evaluation in main-text Section 3.15 now contributes these (ICC 0.55–0.93 across dimensions), revealing a null association between computational E and physician-rated utility
- Requires prospective validation in future studies with clinician panels

S5 Statistical Analysis

S5.1 Database

MIMIC-III PostgreSQL database containing 46,520 ICU admissions with comprehensive clinical data.

Scope of this cohort. The MIMIC-III cohort described in this section was used for *component-level and convergence validation only.* The primary cohort is MIMIC-IV (5,000 ICU patients; prolonged-stay endpoint); all primary-endpoint analyses in the main text (Tables 1–4; Figures 2–4) use MIMIC-IV. Cross-dataset (MIMIC-III↔MIMIC-IV) and external (eICU) analyses are identified as such wherever reported.

S5.2 Patient Cohort

We selected 5,000 critically ill patients through representative sampling from the MIMIC-III database. Selection criteria included:

- Complete demographic and admission data availability
- ICU admission with comprehensive patient characteristics
- Diverse diagnostic categories representing emergency admission scenarios
- Emergency admissions only, ensuring acute care clinical context

S5.3 Feature Engineering

We implemented a comprehensive feature engineering process extracting a mean of 142.4 features per patient (range: 130–155 features depending on data availability) from multiple MIMIC-III data sources:

Step 1: Demographic and Administrative Data

- Patient demographics: age, gender, admission type, admission location, discharge location, insurance, marital status, ethnicity
- ICU characteristics: ICU length of stay (hours), intensive care unit type, prolonged stay indicator ($>$ 72 hours)

Step 2: Vital Signs Aggregation (First 24 Hours)

- **Heart Rate** (item IDs: 211, 220045): mean, minimum, maximum
- **Systolic Blood Pressure** (item IDs: 51, 442, 455, 6701, 220050, 220179): mean, minimum, maximum
- **Diastolic Blood Pressure** (item IDs: 8368, 8440, 8441, 8555, 220051, 220180): mean
- **Temperature** (item IDs: 223761, 678): mean
- **Respiratory Rate** (item IDs: 618, 615, 220210, 224690): mean
- SpO_2 (item IDs: 646, 220277): mean

Step 3: Laboratory Values Aggregation

- Laboratory test counts: unique lab tests, total lab measurements, abnormal lab results count
- Laboratory means: creatinine, potassium, sodium, blood urea nitrogen (BUN), bilirubin, pH, chloride, glucose, hemoglobin, white blood cell count (WBC)

Step 4: Clinical Procedure and Diagnosis Features

- Procedure counts: unique procedures, total procedures, cardiac procedures, vascular procedures
- Diagnosis indicators: unique diagnoses, total diagnoses, diabetes, hypertension, myocardial infarction, heart failure, respiratory disease

Step 5: Medication Features

- Medication counts: unique medications, total medication orders, insulin orders, heparin orders, vasopressor orders

Step 6: Engineered Clinical Features

- Clinical complexity score, hemodynamic instability indicator, lab abnormality rate, organ system involvement score, critical care intensity score, age-adjusted risk score, medication burden, vital stability score

From this comprehensive feature set, the final model used **26 features** selected for optimal balance between predictive performance and clinical interpretability, including enhanced features from chartevents (GCS components, MAP, urine output). One feature (gcs_motor_mean) was dropped during imputation due to all-NaN values (see Confidence Component section for detailed feature list).

S5.4 Data Leakage Prevention

1. **Age Calculation**: Age was calculated at ICU admission time (EXTRACT(YEAR FROM AGE(i. rather than using date of death (dod), which would directly leak mortality information. The original calculation using COALESCE(p.dod, NOW()) created a correlation of 0.889 with mortality; the corrected calculation reduced this to -0.274.

2. **ICU Length of Stay Exclusion**: ICU LOS was excluded from the final model as it is only known after the ICU stay ends, creating temporal leakage.

3. **Temporal Windows**: All clinical data (vital signs, labs) were restricted to the first 24 hours of ICU admission, ensuring features are available at prediction time.

S5.5 Primary Analysis

Trust scores calculated using the optimized weighted formula $T = \alpha \cdot C + \beta \cdot E + \gamma \cdot S$ (first-order linear approximation of the general trust function $T = f(C, E, S)$) with **dataset-dependent optimal weights** ($\alpha = 0.60$, $\beta = 0.24$, $\gamma = 0.16$) for each patient. For the primary prolonged ICU stay endpoint, the mean trust score was 0.364 ± 0.145 with 95% confidence intervals calculated using bootstrap resampling (5,000 iterations). The secondary mortality endpoint yielded a mean trust score of 0.239 ± 0.098. Statistical power analysis confirmed 99% power to detect trust score differences with effect size 0.742.

S6 Reproducibility Protocol

To ensure reproducible trust convergence validation, we implemented:

S6.1 Multiple Independent Executions

Each validation run executed independently with identical parameters, ensuring consistency across different computational environments.

S6.2 Statistical Analysis

Confidence intervals calculated for all trust metrics using both normal approximation and bootstrap methods (5,000 iterations), with significance testing to validate convergence achievement reliability.

S6.3 Ethics and Data Use

This study used existing de-identified data from the MIMIC-III, MIMIC-IV, and eICU Collaborative Research Database, accessed through PhysioNet under the applicable credentialing and data-use requirements. The investigators had no direct interaction with the patients represented in these databases and could not readily ascertain their identities. Under the U.S. Federal Policy for the Protection of Human Subjects (45 CFR §46.102(e)(1)), these secondary analyses did not constitute human-subjects research requiring additional Institutional Review Board approval. Accordingly, additional IRB

approval and individual patient informed consent were not required. The study was conducted in accordance with the applicable ethical principles of the Declaration of Helsinki.

S6.4 Code Availability

Detailed implementation methodology and pseudo-code are provided in Supplementary Methods. Core algorithms and statistical analysis methods are described with sufficient detail for reproduction.

S7 Detailed Component Definitions and Mathematical Formulations

S7.1 Confidence Component (C) — Detailed Specification

S7.1.1 Current Implementation

The Confidence (C) component is defined as the **calibrated confidence in the predicted class**: $C = \max(\hat{p}, 1 - \hat{p})$, where $\hat{p}$ is the isotonic-calibrated positive-class probability, achieving calibration (ECE 0.029 on the primary held-out test cohort). Defining C as $\hat{p}$ itself would penalize confident low-risk predictions; results under both definitions appear in Section S14. This approach focuses on probability calibration—ensuring that predicted probabilities accurately reflect true outcome frequencies.

S7.1.2 Model Training Details

The base prediction model was trained on 4,000 patients (80% of dataset) for in-hospital mortality risk stratification using logistic regression with balanced class weights and liblinear solver. Through comprehensive feature engineering from the MIMIC-III database, we extracted a mean of 142.4 features per patient from multiple data sources, including enhanced chartevents features (see Feature Engineering section).

From this comprehensive feature set, the final logistic regression model used **26 features** selected for optimal predictive performance and clinical interpretability:

Demographics (2 features):

1. **age** (years, calculated at ICU admission time to avoid data leakage)
2. **gender** (binary: 1=male, 0=female)

Vital Signs (12 features):

1. **hr_mean** (mean heart rate, bpm)
2. **sbp_mean** (mean systolic blood pressure, mmHg)
3. **dbp_mean** (mean diastolic blood pressure, mmHg)
4. **resp_mean** (mean respiratory rate, breaths/min)
5. **temp_mean** (mean body temperature, °C)
6. **spo2_mean** (mean oxygen saturation, %)

7. **sbp_min** (minimum systolic blood pressure, mmHg — hemodynamic instability indicator)
8. **map_mean** (mean arterial pressure, mmHg — enhanced from chartevents)
9. **gcs_total_mean** (mean Glasgow Coma Scale total — enhanced from chartevents)
10. **gcs_eye_mean** (mean GCS eye component — enhanced from chartevents)
11. **gcs_verbal_mean** (mean GCS verbal component — enhanced from chartevents)
12. **urine_output_total** (total 24-hour urine output, mL — enhanced from chartevents)

Laboratory Values (5 features):

1. **bun_mean** (mean blood urea nitrogen, mg/dL) — F-score: 139.83
2. **hemoglobin_mean** (mean hemoglobin, g/dL) — F-score: 82.00
3. **creatinine_mean** (mean serum creatinine, mg/dL) — F-score: 43.23
4. **bilirubin_mean** (mean total bilirubin, mg/dL) — F-score: 23.67
5. **potassium_mean** (mean serum potassium, mEq/L) — F-score: 11.96

Lab Metrics (2 features):

1. **unique_lab_tests** (number of unique laboratory tests) — F-score: 27.77
2. **abnormal_labs_count** (count of abnormal lab results) — F-score: 24.01

Comorbidities (4 features):

1. **unique_diagnoses** (number of unique diagnoses) — F-score: 49.95
2. **heart_failure** (heart failure diagnosis indicator) — F-score: 47.24
3. **respiratory_disease** (respiratory disease diagnosis indicator) — F-score: 33.48
4. **diabetes** (diabetes diagnosis indicator) — F-score: 11.54

Note: The feature **vasopressor_orders** was dropped during imputation due to all-NaN values (sparse medication data). The feature **gcs_motor_mean** was also dropped during imputation due to all-NaN values (sparse GCS component data).

S7.1.3 Model Performance

The model achieved an **AUC-ROC of 0.85** (95% CI: 0.82–0.88) on the test set for mortality prediction, representing a significant improvement over the baseline 7-feature model (AUC: 0.68). The primary prolonged ICU stay endpoint achieved AUC 0.80 (95% CI: 0.77–0.83); the mortality endpoint achieved a higher AUC than prolonged stay, reflecting the stronger signal-to-noise for in-hospital mortality in this validation cohort. Enhanced feature engineering incorporating Glasgow Coma Scale (GCS) components, Mean Arterial Pressure (MAP), and urine output contributed to improved predictive performance. Feature importance analysis revealed age as the strongest predictor (F-score: 226.12,

$p < 0.001$), followed by laboratory values (BUN: F-score 139.83, hemoglobin: 82.00, creatinine: 43.23) and comorbidities (unique_diagnoses: F-score 49.95, heart_failure: F-score 47.24). Model probabilities were calibrated using isotonic regression on a 1,000-patient hold-out test set, achieving Expected Calibration Error (ECE) of **0.044**.

Calibration without test-label leakage. Isotonic calibration is fit on five-fold out-of-fold *training* predictions, so the calibration map never sees test labels. Fitting the calibrator on the held-out test set instead would leak test labels into calibration; it changes AUCs by 0.004–0.026 (at most 0.013 on the primary MIMIC-IV cohort, e.g., prolonged-stay LR 0.788 vs. 0.780) without altering any qualitative conclusion.

Test Set Performance (20% hold-out, 1,000 patients):

- **AUC-ROC (mortality)**: 0.85
- **AUC-ROC (prolonged ICU stay, primary endpoint)**: 0.80
- **Calibration Error (ECE)**: 0.044

S7.2 Explainability Component (E) — Detailed Algorithm

S7.2.1 Complete 6-Component Algorithm

Algorithm S1 Calculate_Explainability(explanation_text, features, prediction)

1: $E_{\text{evidence}} \leftarrow \text{Evidence\_Linkage\_Score}(\text{explanation\_text}, \text{features}, \text{threshold} = 0.3)$
2: $E_{\text{coherence}} \leftarrow \text{Reasoning\_Coherence\_Assessment}(\text{explanation\_text}, \text{causal\_terms})$
3: $E_{\text{specificity}} \leftarrow \text{Specificity\_Actionability\_Score}(\text{explanation\_text}, \text{clinical\_keywords})$
4: $E_{\text{conciseness}} \leftarrow \text{Conciseness\_Score}(\text{explanation\_text}, \text{optimal\_length} = 30, \text{penalty} = 0.05)$
5: $E_{\text{counterfactual}} \leftarrow \text{Counterfactual\_Uncertainty\_Assessment}(\text{explanation\_text})$
6: $E_{\text{fidelity}} \leftarrow \text{Model\_Fidelity\_Score}(\text{explanation\_text}, \text{prediction}, n_{\text{perturbations}} = 30)$
7: **return** $\sum_{i=1}^{6}(w_i \times E_i)$ where $w = \{0.25, 0.20, 0.20, 0.15, 0.10, 0.10\}$

S7.2.2 Component Definitions

- E_{evidence} **(25%)**: TF-IDF cosine similarity between explanation and clinical features (threshold: 0.3). Uses English-specific text preprocessing. A BERTScore validation confirms the rank-ordering is preserved ($\rho > 0.6$), and TF-IDF's known limitations (zero score for synonymous terms) do not materially alter deployment decisions (see Section BERTScore Evidence Linkage Validation).
- $E_{\text{coherence}}$ **(20%)**: Causal reasoning term frequency with systematic approach indicators. Relies on English-specific causal terms (e.g., "because", "therefore", "due to").
- $E_{\text{specificity}}$ **(20%)**: Clinical actionability using keyword-based medical terminology validation against hardcoded clinical feature categories. **Note**: Current implementation uses hardcoded dictionaries rather than SNOMED CT integration.
- $E_{\text{conciseness}}$ **(15%)**: Length optimization with $\lambda = 0.05$ penalty for deviations from 30-word target. Assumes English tokenization.

- $E_{\textbf{counterfactual}}$ **(10%)**: Uncertainty quantification through confidence interval analysis. Uses English-specific uncertainty phrases.

- $E_{\textbf{fidelity}}$ **(10%)**: Surrogate model agreement using 30 perturbations with $\alpha = 1.0$ surrogate. **As implemented**: this term measures how well a local linear surrogate reproduces the model's predictions on perturbed inputs; it does not use the explanation text, and because no model function was supplied it equalled 0 in every reported analysis. The conciseness term equals 1 for 10–80 words and decays as $\exp(-0.05\,|\text{words} - 30|)$ only outside that range. **Limitation (disclosed)**: the perturbation strategy in the current implementation is generic (noise-based) rather than clinically structured, which contributes to the near-constant behavior of E observed for some architectures (see main-text Section 3.2); clinically meaningful perturbations and a physician-anchored fidelity score are planned refinements (main-text Section 4).

Note on Generalizability: The current implementation is optimized for English-language ICU/critical care contexts. See Limitations section for detailed discussion of constraints and future enhancement directions.

S7.2.3 Provenance of Supplementary File S2

The case explanations in Supplementary File S2 were generated by a logistic-regression model trained on a comprehensive MIMIC-III feature set—a pipeline separate from the primary MIMIC-IV model, and whose feature set and attribution differ from those of the primary model. Feature contributions in S2 were computed as coefficient × raw (uncentered) feature values, a method that does not adjust for each feature's population mean; regenerated explanations with centered attribution are planned as follow-up work. Temperatures in S2 are recorded in degrees Fahrenheit; one explanation template printed the Fahrenheit value with a Celsius label and described normal or low temperatures as "fever". S2 reproduces the case materials verbatim as rated, and its Instructions sheet lists the known defects.

S7.3 Semantic Similarity Component (S) — Detailed Specification

The Semantic Similarity (S) component achieved a mean score of **0.656** (SD: 0.032) across the validation cohort, reflecting strong alignment between the AI's derived features and human-derived clinical concepts.

S7.3.1 Enhanced Semantic Similarity Calculation

$$S = 0.30 \cdot S_{\text{guideline}} + 0.25 \cdot S_{\text{knowledge}} + 0.20 \cdot S_{\text{reasoning}} + 0.15 \cdot S_{\text{terminology}} + 0.10 \cdot S_{\text{evidence}} \quad \text{(S2)}$$

S7.3.2 Knowledge Sources

- **Clinical Guidelines**: **Current Implementation**: Hardcoded simplified guideline statements for 4 ICU/critical care contexts (sepsis, shock, respiratory failure, cardiac arrest). The guidelines are generic clinical knowledge statements and do not represent actual content from authoritative sources. **Future Work**: Will implement dynamic guideline loading from authoritative sources with versioning support.

- **Terminology**: **Current implementation uses hardcoded Python dictionaries** (5 categories: vital_signs, laboratory_values, clinical_scores, interventions, diagnoses) rather than SNOMED CT/UMLS integration. **Future work**: Will integrate SNOMED CT and UMLS for comprehensive terminology matching.
- **Embedding Model**: sentence-transformers/all-MiniLM-L6-v2 (384-dim) — English-optimized, general-purpose model
- **Similarity Thresholds**: Guideline alignment > 0.7, Terminology match > 0.6, Reasoning coherence > 0.5

S7.3.3 Component Definitions

- $S_{\textbf{guideline}}$ **(30%)**: Cosine similarity between clinical explanation and guideline text using sentence-transformers/all-MiniLM-L6-v2, threshold=0.7. Uses hard-coded guidelines for 4 ICU/critical care contexts.
- $S_{\textbf{knowledge}}$ **(25%)**: Medical knowledge consistency assessment using hardcoded terminology dictionaries. Uses English reasoning indicators.
- $S_{\textbf{reasoning}}$ **(20%)**: Clinical reasoning pattern alignment using structured templates, coherence threshold=0.5. Uses English-specific reasoning patterns.
- $S_{\textbf{terminology}}$ **(15%)**: Medical vocabulary appropriateness using hardcoded terminology dictionaries with English terms.
- $S_{\textbf{evidence}}$ **(10%)**: Evidence-based medicine indicators using English keyword matching for evidence quality phrases.

S7.4 Mathematical Properties and Theoretical Guarantees

Boundedness: The trust score is bounded within $[0, 1]$ by construction:

$$T_{\min} = 0 \cdot \alpha + 0 \cdot \beta + 0 \cdot \gamma = 0 \tag{S3}$$

$$T_{\max} = 1 \cdot \alpha + 1 \cdot \beta + 1 \cdot \gamma = \alpha + \beta + \gamma = 1.0 \tag{S4}$$

Monotonicity: The trust function is monotonically increasing in each component:

$$\frac{\partial T}{\partial C} = \alpha > 0, \quad \frac{\partial T}{\partial E} = \beta > 0, \quad \frac{\partial T}{\partial S} = \gamma > 0 \tag{S5}$$

Linearity and Continuity: The trust formula is linear and continuous, enabling gradient-based optimization, smooth trust dynamics, and computational efficiency in real-time clinical decision support.

Weight Normalization: Component weights satisfy:

$$\alpha + \beta + \gamma = 1.0, \quad \text{with } \alpha, \beta, \gamma \geq 0 \tag{S6}$$

S7.5 Adaptive Trust Threshold Integration

The trust framework integrates the adaptive threshold $\tau(E) = 0.5 + 0.2 \times (1 - E)$ to determine deployment eligibility:

$$\text{Deployment Decision: } T \geq \tau(E) \tag{S7}$$

where:

- $T = \alpha \cdot C + \beta \cdot E + \gamma \cdot S$ represents the trust score for the AI system
- $\tau(E) = 0.5 + 0.2 \times (1 - E)$ represents the adaptive threshold based on explainability
- Systems with $T \geq \tau(E)$ meet the minimum trust requirement for deployment

Extension to Multi-System Orchestration: While validated here on single-model predictions, the framework naturally extends to orchestrating heterogeneous AI systems by applying T scores to select the most trustworthy system per case. The optimization objective would be:

$$\text{maximize: } \sum_{i=1}^{n} T_i \cdot R(S_i) \tag{S8}$$

$$\text{subject to: } T_i \geq \tau_i(E_i) \text{ for all selected systems } i \tag{S9}$$

where $R(S_i) \in [0, 1]$ represents system relevance score for the current clinical context. Future work will validate this extension in multi-system clinical decision-support ecosystems.

S8 Trust Framework Implementation

Our trust framework integrates three specialized components:

S8.1 SimplifiedTrustAnalyzer

Core trust computation engine implementing the weighted trust formula as a first-order linear approximation of the general trust function $T = f(C, E, S)$. Trust scores are calculated as:

$$T = C \times 0.60 + E \times 0.24 + S \times 0.16 \tag{S10}$$

Note: These weights are dataset and context-dependent, optimized for MIMIC-III ICU mortality prediction with logistic regression. Different clinical domains or AI architectures may require re-optimization.

The analyzer determines trust levels (Very High: ≥ 0.8, High: ≥ 0.6, Moderate: ≥ 0.4, Low: < 0.4) and assesses clinical suitability (Clinical deployment ready: ≥ 0.7, Requires validation: ≥ 0.5, Not suitable: < 0.5).

S8.2 ClinicalExplainabilityDashboard

Enhanced explainability scoring system calculating explainability scores through:

- **Feature Usage Analysis**: Base score calculated as $\min(1.0, \text{features\_used}/5.0)$
- **Differential Diagnosis Bonus**: An additional 0.2 bonus applied when predictions include differential diagnoses

S8.3 ClinicalTrustMetrics

Comprehensive trust metadata framework providing trust score, competence, explainability, semantic similarity components, clinical threshold validation, trust level determination, and reliability metrics.

S9 Mathematical Proofs and Convergence Theory

Scope. Monotonicity follows directly from non-negative weights; the online update of Theorem 1 is not exercised by the retrospective evaluation; and the bound of Theorem 3 has not been numerically instantiated. None of these properties is used as evidence for the main-text results.

Our framework implements formal mathematical proofs establishing theoretical foundations for trust assessment:

S9.1 Theorem 1: Trust Calibration Convergence (Robbins-Monro)

Mathematical Statement: Given a sequence of clinical outcomes $\{y_t\}$ and predictions $\{p_t\}$, the calibrated trust function $T_i(t)$ converges to optimal trust calibration T_i^* with probability 1 under Robbins-Monro conditions.

S9.1.1 Required Assumptions

1. **Data Stationarity**: Clinical data distribution remains stable over calibration period
2. **Unbiased Outcome Access**: True clinical outcomes available for calibration without selection bias
3. **Independent Observations**: Patient outcomes are independent
4. **Bounded Gradients**: Calibration loss gradients remain bounded (satisfied by Brier score)

S9.1.2 Detailed Proof

Step 1: Calibration Update Rule Define the calibration update rule:

$$T_i(t+1) = T_i(t) + \eta_t \cdot \nabla L(T_i(t), y_t, p_t) \tag{S11}$$

where L represents the Brier score calibration loss: $L(T_i, y, p) = (p_{\text{calibrated}}(T_i) - y)^2$.

Step 2: Learning Rate Schedule (Robbins-Monro Conditions) Step-size schedule: a schedule such as $\eta_t = 1/\sqrt{t}$ does *not* satisfy the Robbins–Monro conditions, because $\sum_t \eta_t^2 = \sum_t 1/t = \infty$. We therefore use $\eta_t = a/(a+t)$ for $a > 0$, which satisfies both conditions:

- **Divergence condition**: $\sum_{t=1}^{\infty} \eta_t = \infty$
- **Convergence condition**: $\sum_{t=1}^{\infty} \eta_t^2 < \infty$

All references to the online update in this supplement should be read as design specifications for prospective deployment: the present validation is retrospective and does not exercise an online trust update (see revised main-text Section 2.5).

Step 3: Loss Function Properties The Brier score has convexity, unique minimum, and bounded gradients.

Step 4: Convergence Analysis (Robbins-Monro Theorem) By the Robbins-Monro stochastic approximation theorem, under the conditions above, we have almost-sure convergence: $T_i(t) \xrightarrow{a.s.} T_i^*$ as $t \to \infty$.

Verification in Our Framework: The Robbins–Monro conditions are properties of the schedule above; the bounded-gradient and stationarity assumptions hold in the retrospective validation setting, while the online update itself is a design specification not exercised by the current offline validation.

S9.2 Theorem 2: Trust Score Monotonicity

Mathematical Statement: The trust framework's component structure ensures that trust scores increase monotonically with improvements in any component (C, E, or S)—a direct consequence of the non-negative component weights. Monotonicity of a non-negatively weighted sum is a structural property and does not constitute an optimality result.

S9.2.1 Required Assumptions

1. **Component Non-Negativity**: All components (C, E, S) are bounded in $[0, 1]$
2. **Weight Normalization**: Component weights satisfy $\alpha+\beta+\gamma = 1$ with $\alpha, \beta, \gamma \geq 0$
3. **Component Independence**: Components are conditionally independent given the clinical context

S9.2.2 Detailed Proof

Step 1: Monotonicity Property The trust function $T = \alpha \cdot C + \beta \cdot E + \gamma \cdot S$ has partial derivatives:

$$\frac{\partial T}{\partial C} = \alpha > 0, \quad \frac{\partial T}{\partial E} = \beta > 0, \quad \frac{\partial T}{\partial S} = \gamma > 0 \tag{S12}$$

Since all weights are non-negative, trust scores increase monotonically with improvements in any component.

Step 2: Weight Selection via Sensitivity Analysis Remark: a finite grid search does not establish optimality; moreover, the selected weights ($\alpha = 0.60$) lie on the boundary of the searched grid, and the search did not use outcome data (Section S1). The grid search is therefore a weight-selection heuristic, not an optimality proof.

Step 3: Selected Configuration The sensitivity analysis across 162 configurations selected ($\alpha = 0.60, \beta = 0.24, \gamma = 0.16$) for the specific dataset and clinical context; this is the best configuration *within the searched grid*, not a global optimum.

Verification in Our Framework: The monotonicity property is satisfied by construction (non-negative weights); the selected configuration achieved the best performance metrics among all tested configurations in the grid.

S9.3 Theorem 3: Clinical Safety Bounds (PAC-Bayes)

Mathematical Statement: The framework establishes probabilistic safety bounds ensuring adverse event rates remain below clinical thresholds with high confidence through PAC-Bayesian analysis.

S9.3.1 Required Assumptions

1. **i.i.d. Patient Population**: Patients drawn independently from similar distribution
2. **Stable Risk Stratification**: Clinical risk categories remain consistent
3. **Bounded Loss Function**: Clinical loss functions are bounded
4. **Prior Knowledge**: Informative priors available for PAC-Bayesian analysis

S9.3.2 Detailed Proof

Step 1: PAC-Bayesian Framework We apply the PAC-Bayesian theorem to bound the expected risk. For a prior distribution P over trust thresholds and posterior distribution Q learned from data, with probability at least $1 - \delta$:

$$\mathbb{E}_Q[\text{Risk}(\tau)] \leq \mathbb{E}_Q[\hat{\text{Risk}}(\tau)] + \sqrt{\frac{\text{KL}(Q||P) + \ln(1/\delta)}{2n}} \tag{S13}$$

Step 2: Safety Bound Formulation For clinical safety, we require:

$$P(\text{Adverse Event} \mid T_i \geq \tau_{\text{safety}}) \leq \epsilon \tag{S14}$$

Step 3: Risk-Stratified Adaptive Thresholds Define risk-stratified thresholds:

$$\tau_i(E, \text{Risk}, \text{Context}) = \begin{cases} 0.7 & \text{ICU (critical care)} \\ 0.65 & \text{Emergency (time-sensitive)} \\ 0.6 & \text{General Ward (standard care)} \\ 0.5 & \text{Outpatient (routine care)} \end{cases} \tag{S15}$$

Step 4: Bound Framework Using PAC-Bayesian analysis with sample size $n = 5,000$ patients and confidence $\delta = 0.05$ (95% confidence), the theoretical bound ensures $P(\text{unsafe} \mid T_i \geq \tau_{\text{safety}}) \leq \epsilon$ with high confidence. We emphasize that this is a theoretical

framework providing the formal structure for safety bounds; the numerical value of ϵ depends on the chosen prior P and posterior Q specifications, which require domain-specific calibration from clinical safety experts. The key contribution is the formal PAC-Bayesian structure itself, not a specific numerical bound.

S10 Deployment Simulation Methodology

To assess the framework's real-world decision impact, we conducted a simulated deployment analysis comparing status-quo deployment (all AI predictions deployed without oversight) against framework-gated deployment (cases with $T \geq \tau(E)$ deploy autonomously; flagged cases trigger human review).

S10.1 Simulation Design

The simulation is computed directly on the 1,000-patient held-out MIMIC-IV test cohort (prolonged ICU stay endpoint) under the predicted-class confidence definition, with per-patient E/S scoring and leak-free out-of-fold calibration:

- **Pass rate**: 76.5% (765/1,000) of cases satisfied $T \geq \tau(E)$ at the default $\kappa = 0.20$
- **Flagged cases**: 23.5% (235/1,000) fell below the adaptive threshold and were routed to human review
- **Model performance**: AUC-ROC of 0.78 (leak-free out-of-fold calibration), corresponding to an overall model error rate of 22.2% on the test cohort
- **Error concentration**: the flagged group had a substantially higher model error rate (34.5%) than the autonomously deployed group (18.4%), confirming that the trust score concentrates model errors into human review

S10.2 Assumptions and Rationale

1. **Model error rate (22.2%)**: Observed directly on the held-out test cohort (primary endpoint, prevalence 32%).
2. **Deployed vs. flagged error rates (18.4% vs. 34.5%)**: Observed directly on the held-out test set; the flagged group carries nearly twice the error rate, consistent with its lower trust scores.
3. **Human review accuracy (80%)**: A conservative estimate based on published literature on clinician diagnostic accuracy in ICU settings (range: 75–95%). We assume clinicians reviewing flagged cases can identify and correct model errors 80% of the time.
4. **Independence of errors**: We assume errors in flagged cases are independent of human review outcomes. In practice, the most challenging cases may be difficult for both models and clinicians, making the 80% accuracy estimate conservative.
5. **Random-deferral baseline**: a random subset of identical size (235 cases) is flagged for review, so that the trust score's error selection can be compared with review volume alone at matched coverage.

S10.3 Calculation

With the framework: deployed errors = $765 \times 0.184 \approx 141$; flagged errors = $235 \times 0.345 \approx 81$; human corrections = $81 \times 0.80 \approx 64.8$; remaining flagged errors = $81 - 64.8 = 16.2$; total residual errors = $141 + 16.2 = 157.2$, against 222 total errors without oversight (a 29% reduction). At the same review coverage, random deferral leaves 180.3 residual errors on average (95% CI: 171.6–189.2, from 1,000 resampling draws), so trust-score gating averts 23.1 additional errors relative to random selection at identical review workload.

At the default $\kappa = 0.20$ the framework flags 23.5% of predictions for human review, and the flagged group carries nearly double the error rate of the deployed group; gating by the trust score outperforms random deferral at matched coverage, confirming that the framework's error selection adds value beyond the volume of review itself.

S10.4 Sensitivity to Human Review Accuracy

We tested the sensitivity of error reduction to varying human review accuracy:

Table S2: Sensitivity of error reduction to human review accuracy.

**Human Accuracy**	**Corrections**	**Total Errors**	**Error Rate**
70%	779	389	7.8%
75%	835	333	6.7%
80%	891	277	5.5%
85%	946	222	4.4%
90%	1,002	166	3.3%
95%	1,058	110	2.2%

Because the flagged fraction is large (93.3%), human-review accuracy has a substantial effect on the overall error rate (2.2–7.8%): the framework's error reduction scales directly with review quality.

S10.5 Limitations of the Simulation

This simulation is a retrospective estimate anchored to observed validation results, not a prospective clinical trial. Key limitations include:

- The deployed error rate and flagged error rate are estimated from aggregate statistics rather than per-case measurements
- Human review accuracy is assumed constant across cases; in practice, accuracy varies with case complexity and clinician expertise
- The simulation does not account for potential human factors (e.g., automation bias, alert fatigue) that may affect real-world performance
- The 80% human accuracy assumption requires prospective validation in the specific clinical context

Despite these limitations, the simulation provides a transparent, reproducible methodology for estimating the framework's deployment impact and can be refined as prospective data become available.

S11 Failure Mode Analysis: Clinical Case Studies

Representative held-out cases under the predicted-class confidence definition are presented in main-text Section 3.7.

S12 Preliminary Cross-Cohort Analysis within MIMIC-III

Note: This section describes a preliminary internal validation conducted on a 2,000-patient subset of MIMIC-III to assess within-institution temporal generalizability. The definitive cross-dataset validation (MIMIC-III n = 5,000 vs. MIMIC-IV n = 4,778) is reported in Section S13.6 and in the main manuscript. Results here are reported for completeness and to document the framework's development trajectory.

To demonstrate that the trust framework generalizes across different patient populations, we conducted a preliminary cross-cohort validation by splitting a 2,000-patient MIMIC-III subset into two temporally separated sub-cohorts sorted by patient identifier (approximating admission time ordering). Cohort A (n = 1,000, subject IDs 52–27,390, mortality rate 56.2%) and Cohort B (n = 1,000, subject IDs 27,420–99,814, mortality rate 36.3%) represent different patient sub-populations with distinct clinical characteristics.

S12.1 Within-Cohort Performance

Each cohort was independently evaluated using the full trust framework (logistic regression with isotonic calibration, six-component explainability metric, and embedding-based semantic similarity). The framework was trained on 70% of each cohort and evaluated on the remaining 30%.

Table S3: Within-cohort and cross-cohort validation results (positive-class confidence definition, $C = \hat{p}$).

Evaluation	AUC	E	S	T	Pass%
Cohort A (internal)	0.797	0.250	0.661	0.504	26.7%
Cohort B (internal)	0.780	0.251	0.656	0.383	3.7%
Train A → Test B	0.768	—	—	—	—
Train B → Test A	0.772	—	—	—	—

S12.2 Key Findings

1. **Consistent explainability and semantic similarity across cohorts**: The E scores (0.250 vs. 0.251) and S scores (0.661 vs. 0.656) were nearly identical despite different prolonged stay rates, confirming that the framework's non-confidence components are population-robust.

2. **Minimal AUC degradation under cross-evaluation**: The internal average AUC (0.789) dropped only 0.019 to the cross-evaluation average (0.770), demonstrating that the model's predictive performance generalizes across subpopulations. The symmetric performance (A→B: 0.768, B→A: 0.772) confirms no systematic bias.

3. **Different trust score distributions reflect population risk**: Cohort A (higher prolonged stay rate) produced higher mean T (0.504 vs. 0.383), correctly reflecting the higher baseline risk. The adaptive threshold adjusts accordingly: both cohorts use the same $\tau(E)$ function, but the distribution of T relative to τ shifts with population risk.

S12.3 Limitations

This analysis uses a temporal split of a single institution's data, which does not replace validation on truly independent datasets. However, the consistency of E and S across cohorts combined with minimal AUC cross-degradation provides evidence that the framework's mathematical structure is stable across patient subpopulations. External validation on the eICU Collaborative Research Database (∼200 hospitals) has now been completed (see Section S13.7), with average cross-institutional AUC (0.816) comparable to internal mortality validation (0.820) under leak-free calibration.

S13 Secondary Endpoint: Mortality

To demonstrate the framework's applicability across clinical endpoints, we validated the same 5,000-patient cohort on in-hospital mortality as a secondary endpoint. The experimental design and model architecture were identical to the primary analysis, with only the target variable changed from prolonged ICU stay (> 72 hours) to mortality.

S13.1 Results

Under out-of-fold calibration with the imputer fit on training data, the mortality AUCs were 0.862 (LR), 0.877 (GB), and 0.829 (MLP), and the prolonged-stay AUCs 0.780 (LR), 0.806 (GB), and 0.765 (MLP) (main-text Table 2). The explainability (E) and semantic similarity (S) component scores were nearly identical between endpoints:

- Prolonged stay: $E = 0.250$, $S = 0.656$
- Mortality: $E = 0.250$, $S = 0.656$

This consistency is expected because both endpoints use the same model architecture and explanation generation method. The trust score distributions differed due to different confidence distributions: the mortality model produced **lower** mean T (0.239, SD: 0.098, vs. 0.364, SD: 0.145, for prolonged stay), reflecting the lower calibrated positive-class probability at the mortality model's lower-prevalence operating point (in-hospital mortality prevalence ∼11% vs. 32% for prolonged stay), under the positive-class confidence definition ($C = \hat{p}$; under the predicted-class definition $C = \max(\hat{p}, 1 - \hat{p})$, mean trust scores rise and the ordering reflects predicted-class confidence instead). The explainability and semantic similarity scores were nearly identical across endpoints.

S13.2 Comparison

The similarity of E and S scores across endpoints confirms that the framework's non-confidence components are endpoint-agnostic: they depend on the explanation quality and semantic alignment, not on the specific clinical outcome being predicted. The C

(confidence) component naturally varies with endpoint because different clinical outcomes have different base rates and model discriminability. This is a feature of the framework's modular design: institutions can substitute their own prediction models and endpoints while maintaining the same trust governance infrastructure.

S13.3 Data and Validation Limitations

This study used retrospective data from MIMIC-IV (primary cohort) with MIMIC-III used for cross-dataset validation; both originate from the same institution and may not fully represent diverse healthcare settings. The 5,000-patient primary cohort represents a single academic medical center's ICU population and may have limited generalizability to other clinical contexts, patient populations, or healthcare systems.

S13.4 Component Metric Limitations

Explainability assessment: The explainability metric was validated using objective computational methods and anti-gaming robustness testing. A completed prospective physician evaluation (n=50 cases, 3 raters) found no correlation between computational E and physician-rated Overall Clinical Utility ($r = -0.15$, $p = 0.31$), with moderate-to-excellent inter-rater reliability (ICC 0.55–0.93 across six of seven dimensions). A systematic inversion was identified: the TF-IDF evidence linkage sub-component rewarded verbatim feature mention over clinical correctness, producing inflated scores for factually misleading explanations. Recalibration through ontology-grounded semantic matching (e.g., SNOMED CT, UMLS) is required before deployment.

Explainability Generalizability Limitations: The current explainability implementation has several generalizability constraints:

1. **Language-Specific Implementation**: All pattern matching, keyword detection, and text processing assumes English. This limits applicability to non-English clinical contexts.
2. **Medical Terminology Hardcoded**: Clinical feature categories are hardcoded for ICU/critical care contexts and may not generalize to other specialties.
3. **TF-IDF Threshold Sensitivity**: The evidence linkage threshold (0.3) may not generalize across different explanation styles or clinical domains.
4. **Fidelity Computation Placeholder**: The local fidelity computation uses placeholder perturbation strategies rather than clinically meaningful feature variations.
5. **Cultural/Regional Variations**: The current implementation assumes Western medicine reasoning patterns.

Future Enhancement Directions:

- Multi-language support through language-specific pattern libraries and multilingual embeddings
- Domain adaptation framework with medical ontology integration (SNOMED CT, UMLS)
- Robust tokenization using language-aware tokenizers

- Advanced fidelity methods with domain-specific perturbation strategies
- Cross-cultural validation

Semantic Similarity Generalizability Limitations: The current semantic similarity implementation has several generalizability constraints:

1. **Language-Specific Implementation**: All components assume English-language clinical text. The embedding model is English-optimized.
2. **Hardcoded Clinical Guidelines**: Guidelines are hardcoded for only 4 ICU/critical care contexts, with no support for other specialties or dynamic guideline loading.
3. **Simplified Medical Terminology Dictionary**: Medical terms are stored as simple dictionaries rather than integrated with medical ontologies (SNOMED CT, UMLS).
4. **General-Purpose Embeddings**: The embedding model is general-purpose rather than medical domain-specific.
5. **Evidence Quality Assessment Limitations**: Evidence quality indicators are English-only phrases, and quality assessment is based on simple keyword matching rather than actual citation parsing.

Future Enhancement Directions:

- Multi-language support through multilingual sentence-transformers
- Medical ontology integration (SNOMED CT, UMLS) for comprehensive terminology matching
- Dynamic guideline loading from authoritative sources with versioning support
- Domain-specific embeddings (BioBERT, ClinicalBERT, PubMedBERT)
- Enhanced evidence assessment with citation parsing and evidence database integration

Confidence calibration: Per-instance calibration performance was evaluated computationally and validated on a hold-out test set. Prospective validation with actual clinical outcomes is needed to confirm real-world calibration performance. The current definition focuses on probability calibration (ECE: 0.044) but does not incorporate prediction uncertainty, data quality, or model stability—these represent important directions for future enhancement.

S13.5 BERTScore Evidence Linkage Validation

To assess whether TF-IDF cosine similarity (the primary implementation) adequately captures semantic similarity between explanations and clinical features, we conducted a comparative validation using BERTScore [1], an embedding-based metric that captures semantic meaning beyond exact token overlap.

Ten test pairs spanning three categories were evaluated: (1) semantic equivalence (synonymous clinical terms, e.g., “tachycardia” vs. “elevated heart rate”), (2) paraphrased

clinical reasoning (e.g., "acute kidney injury" vs. "elevated creatinine indicating renal impairment"), and (3) non-matching controls (unrelated clinical concepts). TF-IDF cosine similarity and BERTScore (using microsoft/deberta-xlarge-mnli) were computed for each pair.

Results confirmed that TF-IDF fails on semantically equivalent but lexically distinct pairs: scores ranged from 0.000 for synonymous terms (e.g., "hypotension" vs. "low blood pressure") to 0.042 for paraphrased clinical statements. BERTScore captured these semantic relationships substantially better, achieving 0.532–0.711 for synonymous terms and 0.481 for paraphrased reasoning. Both methods correctly assigned low scores to non-matching controls (TF-IDF: 0.000; BERTScore: 0.120). Importantly, the rank ordering of E_{evidence} across the 5,000-patient cohort was preserved between the two methods (Spearman $\rho > 0.6$), indicating that TF-IDF's semantic blind spots do not materially alter the framework's deployment decisions. BERTScore serves as a complementary validation confirming that the primary TF-IDF implementation is adequate for discrimination while remaining computationally efficient.

S13.6 Cross-Dataset Validation: MIMIC-III vs. MIMIC-IV

To address generalizability concerns, we conducted a four-scenario cross-dataset validation between MIMIC-III (n = 5,000) and MIMIC-IV (n = 4,778). Both datasets originated from Beth Israel Deaconess Medical Center (BIDMC) but cover different time periods (MIMIC-III: 2001–2012; MIMIC-IV: 2008–2022) and use different database schemas. Feature definitions were aligned across datasets using a unified 36-feature pipeline with matched mortality outcomes (in-hospital mortality via `hospital_expire_flag`).

Methodological safeguards: (1) Isotonic regression calibrated on training set only (no test-label leakage); (2) per-patient E and S scores for 200 randomly sampled test patients per scenario; (3) class-balanced sample weights for all models; (4) feature-based explanations with clinical context for all model types.

Results: The average cross-dataset AUC (0.846) was comparable to the average internal AUC (0.836), with a difference of only 0.011—well below the 0.05 threshold for excellent generalizability. Best cross-dataset models: MIMIC-III→MIMIC-IV MLP (AUC 0.849, 95% CI: 0.833–0.866) and MIMIC-IV→MIMIC-III MLP (AUC 0.843, 95% CI: 0.825–0.860).

Demographic comparison:

Table S4: Demographic comparison of MIMIC-III and MIMIC-IV cohorts.

**Variable**	**MIMIC-III** (n = 5,000)	**MIMIC-IV** (n = 4,778)
Age, mean (SD)	62.1 (16.5)	63.4 (16.1)
Male, n (%)	2,840 (56.8%)	2,705 (56.6%)
Mortality, n (%)	562 (11.2%)	551 (11.5%)
Emergency admit, n (%)	4,168 (83.4%)	3,159 (66.1%)
Diabetes, n (%)	1,700 (34.0%)	2,165 (45.3%)
Hypertension, n (%)	2,100 (42.0%)	2,626 (55.0%)
Heart rate, mean (SD)	85.5 (15.7)	84.9 (16.1)
SBP, mean (SD)	119.3 (16.8)	117.9 (16.4)
SpO_2, mean (SD)	97.1 (2.6)	96.8 (2.1)
Temperature, mean (SD)	98.1 (3.4)	98.3 (0.9)

Component stability across datasets: E scores: logistic regression (LR) 0.38, gradient boosting (GB) 0.38, multilayer perceptron (MLP) 0.25 (constant fallback). S scores: 0.655–0.661 across all scenarios. The consistency of S across datasets confirms population-robustness of the semantic alignment component.

Known limitation: Both MIMIC datasets originate from the same institution (BIDMC). To address this, we performed external validation on the eICU Collaborative Research Database (see Section S13.7 below).

S13.7 External Cross-Institutional Validation: MIMIC-IV vs. eICU

Note. Results in this subsection use the positive-class confidence definition ($C = \hat{p}$); results under the predicted-class definition are in main-text Tables 2–4 and Section S14.

To assess true external generalizability beyond a single institution, we performed cross-dataset validation between MIMIC-IV ($n = 5{,}000$, primary cohort) and the eICU Collaborative Research Database ($n = 5{,}000$, randomly sampled from >200,000 ICU stays across approximately 200 US hospitals). eICU uses a fundamentally different database schema from MIMIC: time offsets are in minutes from ICU admission (not timestamps), vital signs are stored in `vitalperiodic` and `vitalaperiodic` tables (not `chartevents`), laboratory values use text labels (not numeric item IDs), and diagnoses use free-text strings (not ICD codes).

Feature mapping: We mapped eICU features to the same 36-feature pipeline used for MIMIC, including non-invasive blood pressure from `vitalaperiodic.noninvasivesystolic` (using COALESCE to prefer invasive measurements where available), reducing SBP missingness from 79% to 3.4%. All clinical data were restricted to the first 24 hours of ICU admission.

Cohort characteristics:

Table S5: Demographic comparison of MIMIC-IV and eICU cohorts.

**Variable**	**MIMIC-IV** ($n = 5{,}000$)	**eICU** ($n = 5{,}000$)
Age, mean (SD)	65.0 (16.5)	63.2 (16.9)
Male, n (%)	2,800 (56.0%)	2,735 (54.7%)
Mortality, n (%)	545 (10.9%)	458 (9.2%)
Prolonged stay (>72h), n (%)	1,600 (32.0%)	1,281 (25.6%)

Results: Under leak-free out-of-fold calibration, the average cross-institutional AUC (0.816) was comparable to the average internal mortality AUC (0.820). Internal MIMIC-IV AUC: 0.83–0.87 (mortality), 0.75–0.80 (prolonged stay). Internal eICU AUC: 0.70–0.84 (mortality), 0.55–0.77 (prolonged stay). Cross-institutional transfer: MIMIC-IV→eICU (LR 0.812, GB 0.807, MLP 0.776), eICU→MIMIC-IV (LR 0.845, GB 0.842, MLP 0.813).

Component stability: E scores: 0.248–0.400; S scores: 0.647–0.664. The minimal variation across datasets confirms that the trust framework's component behavior is population-robust.

Data quality note: Temperature was missing for 91.7% of eICU patients (stored in `nursecharting` rather than `vitalperiodic`); handled via median imputation. A future iteration should extract temperature from `nursecharting` for complete feature coverage.

Interpretation: The comparable AUCs across completely different hospital systems (single-institution vs. ∼200 hospitals) and data infrastructures (different schemas, coding

conventions, and time representations) demonstrate robust external generalizability. This directly addresses the concern that validation was limited to a single institution.

S13.8 Threshold Discriminability: Full Sensitivity Analysis

Note. Results in this subsection use the positive-class confidence definition ($C = \hat{p}$); results under the predicted-class definition are in main-text Tables 2–4 and Section S14.

Kappa sweep: Pass rates as a function of κ ($\theta_0 = 0.5$ fixed) across all 12 scenario-model combinations (positive-class confidence definition, $C = \hat{p}$; predicted-class values appear in main-text Table 2):

Table S6: Pass rates at selected κ values across scenarios and models.

Scenario	Model	$\kappa = 0.10$	$\kappa = 0.20$	$\kappa = 0.30$	$\kappa = 0.40$
MIMIC-III→IV	LR	2.68%	0.94%	0.65%	0.33%
MIMIC-III→IV	GB	7.26%	6.74%	5.59%	2.60%
MIMIC-III→IV	MLP	3.81%	3.68%	1.44%	0.00%
MIMIC-IV→III	LR	1.66%	1.62%	0.00%	0.00%
MIMIC-IV→III	GB	6.18%	6.18%	4.88%	2.94%
MIMIC-IV→III	MLP	3.48%	2.04%	1.10%	0.00%
MIMIC-III internal	LR	2.73%	1.13%	1.00%	1.00%
MIMIC-III internal	GB	7.27%	7.20%	5.93%	3.00%
MIMIC-III internal	MLP	2.47%	2.47%	1.00%	0.00%
MIMIC-IV internal	LR	3.07%	1.81%	0.35%	0.00%
MIMIC-IV internal	GB	7.04%	6.69%	5.37%	4.11%
MIMIC-IV internal	MLP	2.86%	2.37%	0.21%	0.00%

Pass rates decrease monotonically with increasing κ (main-text Figure 5), confirming that $\tau(E)$ provides tunable gatekeeping. The 2D sensitivity surface (main-text Figure 2) visualizes the joint effect of (κ, θ_0) on pass rates.

Flagged case profiling: Flagged cases differed from passed cases in confidence and trust score across all scenarios. Representative results (MIMIC-III→MIMIC-IV, Gradient Boosting): C flagged 0.034 vs. passed 0.237 ($p < 0.001$); T flagged 0.206 vs. passed 0.515 ($p < 0.001$).

S13.9 Linear vs. Nonlinear Trust Formulation: Full Results

Note. Results in this subsection use the positive-class confidence definition ($C = \hat{p}$); results under the predicted-class definition are in main-text Tables 2–4 and Section S14.

Across all 12 scenario-model combinations:

Linear vs. Interaction: 100% decision agreement (0 disagreements in any scenario). Interaction coefficients for LR and GB models were numerically zero ($< 10^{-13}$), confirming no detectable interaction effects. For MLP, small C×E (0.10) and C×S (0.26) coefficients were estimated but produced zero decision disagreements. Pearson $r(L, I) = 1.0000$ for all scenarios.

Linear vs. Multiplicative: Decision agreement 97.8–100%. Maximum disagreements: 107 cases (MLP, MIMIC-III→MIMIC-IV). Pearson $r(L, M)$: 0.960–0.985. The

multiplicative formulation systematically lowers T (because C^α with $C \ll 1$ produces smaller values), increasing flagging rates but not qualitatively changing the governance landscape.

Interpretation: The linear additive formulation showed high decision agreement with the alternatives in these scenarios. Because the interaction coefficients were estimated by regressing on the linear trust score itself, the zero coefficients and perfect agreement are partly circular; an outcome-anchored comparison under the predicted-class definition is reported in main-text Table 3.

S13.10 S-Component Ablation: Full Results

Note. Results in this subsection use the positive-class confidence definition ($C = \hat{p}$); results under the predicted-class definition are in main-text Tables 2–4 and Section S14.

Across all 12 scenario-model combinations, removing S and renormalizing ($\alpha' = 0.714$, $\beta' = 0.286$):

Decision agreement: 98.7–100%. Maximum disagreements: 65 cases (GB, MIMIC-IV→MIMIC-III, all gained by S). No cases were lost by including S.

Trust score reduction: Mean $\Delta T = T_{\text{full}} - T_{\text{no-S}}$ ranged from 0.074 to 0.084 across scenarios. Pearson $r(T_{\text{full}}, T_{\text{no-S}}) = 1.000$ for all scenarios (perfect linear correlation because S adds a nearly constant offset).

Interpretation: S contributes a measurable trust score increment ($\approx$0.08, or approximately one-third of the standard deviation of T) and is intended to capture guideline alignment that C and E cannot measure. In the current low-prevalence setting where most T values are far from τ, S rarely changes deployment decisions. However, in higher-prevalence settings or with better-calibrated models, S's discriminative contribution would increase. S is retained as a structural slot for authoritative guideline grounding; in this cohort its discriminative contribution is negligible (trust scores with and without S correlate at $r = 1.000$; main-text Section 3.14).

S13.11 Framework Assumptions

The mathematical framework assumes:

1. Component independence (violations may affect trust score interpretation)
2. Weight stability across clinical contexts (may require context-specific recalibration)
3. Linear additive combination appropriateness (non-linear relationships may exist)
4. Temporal stability of clinical reasoning patterns (may change with evolving medical knowledge)

S13.12 Deployment Readiness

While this framework provides a methodological foundation, clinical deployment requires additional validation including:

1. Prospective clinical outcome correlation studies
2. Clinician-in-the-loop validation of trust score interpretability and actionability

3. Integration testing with existing clinical workflows
4. Regulatory compliance validation for specific clinical applications

S13.13 Generalizability Constraints

The framework was developed and validated within specific computational and clinical constraints that may limit generalizability:

1. Reliance on structured electronic health record (EHR) data may limit applicability to settings with limited data infrastructure
2. English-language clinical reasoning patterns may not generalize to other languages or cultural clinical practice patterns
3. ICU emergency admission focus may require adaptation for outpatient, elective, or specialty clinical contexts
4. Multi-center validation (MIMIC-IV, MIMIC-III, and eICU from approximately 200 hospitals) may still not reflect all diverse healthcare system patterns or patient populations; validation in non-US healthcare systems remains needed

S13.14 Theoretical Limitations

The mathematical convergence guarantees hold under specific assumptions (data stationarity, unbiased outcome access, clinical guideline stability) that may be violated in real-world clinical environments with concept drift, evolving medical knowledge, or institutional practice variations.

S13.15 Future Work

Clinical Validation — Phase 1 Complete: A prospective physician evaluation was completed in June 2026 (n=50 cases, 3 critical care physicians, 7-dimension Likert rating). The primary endpoint — Pearson correlation between computational E and physician-rated Overall Clinical Utility — was not met ($r = -0.15$, $p = 0.31$). Inter-rater reliability was moderate-to-excellent on six of seven dimensions (ICC 0.55–0.93), indicating that the null result is not explained by rating noise; because the rated explanations contained generator errors and raters were not blinded, the study cannot separate a defective metric from defective explanations (main-text Section 2.12). A systematic inversion was identified: cases designed as "Poor" quality ($E_{\text{target}} = 0.25$) achieved the highest computational scores ($E = 0.443$) while receiving the lowest physician ratings ($\bar{U} = 1.00/5$), because the TF-IDF sub-component rewards feature verbatim mention over clinical correctness. Subsequent phases are:

- **Phase 1b (immediate)**: Recalibrate E using ontology-grounded semantic matching (SNOMED CT or UMLS alignment) to replace TF-IDF evidence linkage; re-validate against physician ratings
- **Phase 2**: Prospective clinical integration study with pilot deployment in a single healthcare institution using the recalibrated metric

- **Phase 3**: Multi-institutional validation across 3–5 healthcare systems (partially initiated: eICU external validation complete with cross-institutional AUC comparable to internal validation; see Section S13.7)

Component Weight Optimization: The default weights ($\alpha = 0.60, \beta = 0.24, \gamma = 0.16$) are policy choices; an outcome-based re-run selected confidence alone (Section S14). Future work should:

1. Refine weights based on specific clinical contexts through clinical validation feedback
2. Investigate joint optimization of the adaptive threshold parameter κ and component weights
3. Validate weight generalizability across multiple datasets and clinical domains

Note on linearity assumption: Under the predicted-class confidence definition, an interaction model fitted to prediction correctness and a multiplicative score rank predictions identically to the linear score (main-text Table 3); the formulations differ only in scale.

Confidence Component Enhancement: Future work will explore enhanced confidence definitions including uncertainty-quantified confidence, multi-component confidence, data quality-adjusted confidence, ensemble-based confidence, epistemic vs. aleatoric uncertainty separation, and stability-based confidence.

Temporal Stability and Long-Term Validation: Future work should assess framework performance stability over time, adaptation to evolving clinical guidelines, and performance in dynamic clinical environments.

S14 Additional Analyses and Results under the Positive-Class Confidence Definition

All analyses in this section use the predicted-class confidence definition $C = \max(\hat{p}, 1-\hat{p})$ unless labelled otherwise, isotonic calibration fit on five-fold out-of-fold training predictions, median imputation fit on training data only, a fixed random seed (42) for splits and models, and S computed with sentence-transformer embeddings (all-MiniLM-L6-v2). Scripts: `r2_primary_analyses.py`, `r2_scenarios.py`, `primary_threshold_figures.py`, and `r2_figure_risk_coverage.py` in the code repository.

S14.1 Primary-Cohort Features

The primary MIMIC-IV model uses 36 features from the first 24 hours of the ICU stay: `age`, `gender`, `emergency_admit`, `hr_mean`, `hr_min`, `hr_max`, `hr_std`, `sbp_mean`, `sbp_min`, `sbp_max`, `dbp_mean`, `resp_mean`, `temp_mean`, `spo2_mean`, `creatinine_mean`, `potassium_mean`, `sodium_mean`, `bun_mean`, `bilirubin_mean`, `ph_mean`, `chloride_mean`, `glucose_mean`, `hemoglobin_mean`, `wbc_mean`, `unique_lab_tests`, `abnormal_labs_count`, `unique_diagnoses`, `diabetes`, `hypertension`, `mi`, `heart_failure`, `respiratory_disease`, `unique_medications`, `insulin_orders`, `heparin_orders`, `vasopressor_orders`. (The 26-feature description in Section S5.3 refers to the MIMIC-III development pipeline.)

S14.2 Outcome-Based Weight Re-optimization

The weight search was re-run on the MIMIC-IV training split (n = 4,000) with the stated objective $L = 0.4\,\mathrm{MAE}(T, T_{\mathrm{target}}) + 0.3\,\mathrm{ECE} + 0.2\,\mathrm{FAR} + 0.1\,\mathrm{FRR}$, where T_{target} is 1 for correctly classified and 0 for misclassified patients, ECE is computed for T against correctness (10 bins), FAR is the proportion of misclassified patients with $T \geq \tau(E)$, and FRR the proportion of correctly classified patients with $T < \tau(E)$. The grid covered the simplex in steps of 0.05 (231 configurations); no metric was normalized and no noise was added. Because the calibration map was fit on the same out-of-fold training predictions, training-set ECE is optimistic; selection was therefore checked on the held-out test set (second table).

Table S7: Weight search on the training split: five best configurations and the default weights.

Weights (α, β, γ)	L	MAE	ECE	FAR	FRR
(1.00, 0.00, 0.00)	0.2747	0.320	0.000	66.8%	13.1%
(0.95, 0.00, 0.05)	0.2786	0.325	0.007	66.8%	13.1%
(0.90, 0.00, 0.10)	0.2824	0.329	0.014	66.8%	13.1%
(0.95, 0.05, 0.00)	0.2858	0.333	0.020	66.8%	13.1%
(0.85, 0.00, 0.15)	0.2862	0.333	0.021	66.8%	13.1%
(0.60, 0.24, 0.16) default	0.3300	0.394	0.114	60.7%	16.6%

Table S8: Held-out test evaluation (n = 1,000) of the default and re-optimized weights.

Weights	Pass rate	L	ECE	FAR	FRR	AURC
Default (0.60, 0.24, 0.16)	76.5%	0.343	0.120	63.5%	19.8%	0.1412
Re-optimized (1, 0, 0)	81.3%	0.302	0.023	73.0%	16.3%	0.1401

S14.3 Same-Threshold Multiplicative Agreement and κ Sweep (18 Combinations)

Table S9: Linear versus multiplicative score applied to the same threshold $\tau(E)$, and AURC of T and of C alone (predicted-class definition). Pass rates in %.

Scenario	Model	Linear pass	Mult. pass	Agreement	Cohen's κ	AURC T	AURC C
MIMIC-IV internal (mortality)	LR	85.4	52.5	67.1	0.32	0.0243	0.0243
MIMIC-IV internal (mortality)	GB	89.1	84.5	95.4	0.80	0.0222	0.0222
MIMIC-IV internal (mortality)	MLP	75.4	67.7	92.3	0.81	0.0317	0.0317
MIMIC-IV internal (prolonged)	LR	37.4	1.8	64.4	0.06	0.1419	0.1419
MIMIC-IV internal (prolonged)	GB	39.1	0.5	61.3	0.01	0.1230	0.1230
MIMIC-IV internal (prolonged)	MLP	60.1	60.1	100.0	1.00	0.1569	0.1569
eICU internal (mortality)	LR	88.1	50.9	62.9	0.25	0.0274	0.0274
eICU internal (mortality)	GB	80.5	48.2	67.7	0.37	0.0252	0.0252
eICU internal (mortality)	MLP	88.8	72.1	83.3	0.49	0.0364	0.0364
eICU internal (prolonged)	LR	46.1	6.7	60.5	0.15	0.1207	0.1207
eICU internal (prolonged)	GB	48.7	4.1	55.3	0.09	0.1213	0.1213
eICU internal (prolonged)	MLP	86.5	0.0	13.5	0.00	0.2364	0.2364
MIMIC-IV → eICU (mortality)	LR	90.6	52.5	61.9	0.21	0.0295	0.0295
MIMIC-IV → eICU (mortality)	GB	80.4	71.5	91.2	0.76	0.0331	0.0331
MIMIC-IV → eICU (mortality)	MLP	97.6	96.7	99.0	0.83	0.0355	0.0355
eICU → MIMIC-IV (mortality)	LR	80.0	51.6	71.6	0.42	0.0413	0.0413
eICU → MIMIC-IV (mortality)	GB	70.4	36.4	66.0	0.39	0.0382	0.0382
eICU → MIMIC-IV (mortality)	MLP	79.3	1.0	21.6	0.01	0.0461	0.0461

Table S10: Pass rate (%) as a function of κ ($\theta_0 = 0.5$), predicted-class definition.

Scenario	Model	$\kappa = 0.0$	$\kappa = 0.1$	$\kappa = 0.2$	$\kappa = 0.3$	$\kappa = 0.4$	$\kappa = 0.5$
MIMIC-IV internal (mortality)	LR	96.5	90.5	85.4	54.9	0.0	0.0
MIMIC-IV internal (mortality)	GB	98.1	94.8	89.1	70.9	58.0	0.0
MIMIC-IV internal (mortality)	MLP	99.9	95.1	75.4	60.5	0.0	0.0
MIMIC-IV internal (prolonged)	LR	91.1	78.9	37.4	14.9	0.0	0.0
MIMIC-IV internal (prolonged)	GB	84.6	61.1	39.1	7.1	0.0	0.0
MIMIC-IV internal (prolonged)	MLP	100.0	82.3	60.1	35.9	0.0	0.0
eICU internal (mortality)	LR	99.3	92.6	88.1	61.7	0.0	0.0
eICU internal (mortality)	GB	99.0	93.4	80.5	61.3	0.0	0.0
eICU internal (mortality)	MLP	95.7	93.3	88.8	51.2	0.0	0.0
eICU internal (prolonged)	LR	95.0	74.1	46.1	14.3	0.0	0.0
eICU internal (prolonged)	GB	99.3	66.0	48.7	4.1	0.0	0.0
eICU internal (prolonged)	MLP	99.9	93.6	86.5	0.0	0.0	0.0
MIMIC-IV → eICU (mortality)	LR	97.6	91.4	90.6	68.2	0.0	0.0
MIMIC-IV → eICU (mortality)	GB	96.0	94.9	80.4	69.9	39.8	0.0
MIMIC-IV → eICU (mortality)	MLP	100.0	99.6	97.6	94.8	90.4	0.0
eICU → MIMIC-IV (mortality)	LR	94.3	88.0	80.0	54.2	0.0	0.0
eICU → MIMIC-IV (mortality)	GB	93.5	80.8	70.4	37.3	0.0	0.0
eICU → MIMIC-IV (mortality)	MLP	97.1	93.5	79.3	38.9	0.0	0.0

S14.4 Risk-Controlled Deployment: All Scenarios

The certified threshold (main-text Section 2.13) deploys a prediction when $C \geq \lambda$; λ is the smallest value on the grid $\{0.50, 0.52, \ldots, 1.00\}$ for which an exact binomial test rejects the hypothesis that the error rate among deployed calibration predictions exceeds α, at level $\delta/26$ with $\delta = 0.05$. With probability at least $1-\delta$ over the calibration sample, the error rate among deployed predictions for exchangeable new patients is at most α. Each test set was split at random into calibration and evaluation halves (200 splits per combination; 1,000 for the primary cohort). On simulated data with a known error rate, the procedure exceeded α in at most 0.3% of 2,000 calibration draws per α.

Table S11: Mean share of predictions deployed by the certified threshold at error budgets $\alpha = 0.10$, 0.15, and 0.20, and the largest share of splits (over the three budgets) in which the error rate among deployed evaluation predictions exceeded α.

Scenario	Model	Model error rate	Deployed, $\alpha = 0.10$	Deployed, $\alpha = 0.15$	Deployed, $\alpha = 0.20$	Max splits with risk $> \alpha$
MIMIC-IV internal (mortality)	LR	9.6%	89.5%	99.9%	100.0%	1.5%
MIMIC-IV internal (mortality)	GB	8.7%	93.4%	100.0%	100.0%	0.0%
MIMIC-IV internal (mortality)	MLP	10.2%	85.6%	99.8%	100.0%	0.5%
MIMIC-IV internal (prolonged)	LR	22.7%	0.0%	0.2%	27.6%	1.0%
MIMIC-IV internal (prolonged)	GB	22.9%	0.1%	24.8%	56.8%	1.0%
MIMIC-IV internal (prolonged)	MLP	24.2%	0.0%	0.3%	15.2%	1.5%
eICU internal (mortality)	LR	9.1%	92.0%	100.0%	100.0%	1.5%
eICU internal (mortality)	GB	8.7%	94.2%	100.0%	100.0%	1.0%
eICU internal (mortality)	MLP	8.5%	93.1%	100.0%	100.0%	1.5%
eICU internal (prolonged)	LR	22.9%	1.1%	22.7%	68.2%	0.5%
eICU internal (prolonged)	GB	22.7%	0.5%	24.0%	62.6%	1.5%
eICU internal (prolonged)	MLP	28.1%	0.0%	0.0%	0.0%	0.0%
MIMIC-IV → eICU (mortality)	LR	8.9%	97.0%	100.0%	100.0%	0.5%
MIMIC-IV → eICU (mortality)	GB	11.4%	91.7%	100.0%	100.0%	0.0%
MIMIC-IV → eICU (mortality)	MLP	9.1%	97.2%	100.0%	100.0%	1.0%
eICU → MIMIC-IV (mortality)	LR	9.7%	93.8%	100.0%	100.0%	0.0%
eICU → MIMIC-IV (mortality)	GB	13.5%	86.5%	94.2%	100.0%	0.0%
eICU → MIMIC-IV (mortality)	MLP	10.2%	93.2%	100.0%	100.0%	0.5%

S14.5 Validation Scenarios under the Positive-Class Confidence Definition

The following tables report the validation scenarios with confidence defined as the calibrated positive-class probability ($C = \hat{p}$). In the first table the isotonic calibrator was fit on the test set, so its AUCs are optimistic by 0.004–0.026; the second compares the two confidence definitions with out-of-fold calibration (imputer fit on the full cohort); in the third, the interaction model was fitted against the linear score itself, so its agreement is partly circular. Main-text Tables 2–4 report the corresponding analyses under the predicted-class definition.

Table S12: Validation scenarios, positive-class definition (calibrator fit on the test set).

Scenario	Model	AUC	E	S	T (mean)	Pass %	N (test)
MIMIC-IV internal (mortality)	LR	0.870	0.252	0.657	0.231	0.7%	1,500
MIMIC-IV internal (mortality)	GB	0.885	0.350	0.649	0.253	2.5%	1,500
MIMIC-IV internal (mortality)	MLP	0.837	0.299	0.664	0.243	0.4%	1,500
MIMIC-IV internal (prolonged)	LR	0.788	0.249	0.657	0.357	3.7%	1,500
MIMIC-IV internal (prolonged)	GB	0.810	0.250	0.657	0.357	3.9%	1,500
MIMIC-IV internal (prolonged)	MLP	0.763	0.300	0.664	0.370	9.1%	1,500
eICU internal (mortality)	LR	0.832	0.250	0.657	0.220	0.1%	1,500
eICU internal (mortality)	GB	0.855	0.250	0.656	0.220	0.3%	1,500
eICU internal (mortality)	MLP	0.727	0.299	0.664	0.233	0.3%	1,500
eICU internal (prolonged)	LR	0.773	0.250	0.657	0.319	0.3%	1,500
eICU internal (prolonged)	GB	0.770	0.250	0.656	0.319	0.5%	1,500
eICU internal (prolonged)	MLP	0.558	0.250	0.664	0.320	0.0%	1,500
MIMIC-IV to eICU	LR	0.820	0.249	0.657	0.220	0.0%	5,000
MIMIC-IV to eICU	GB	0.814	0.350	0.647	0.242	0.8%	5,000
MIMIC-IV to eICU	MLP	0.787	0.400	0.657	0.256	0.0%	5,000
eICU to MIMIC-IV	LR	0.849	0.254	0.657	0.231	0.0%	5,000
eICU to MIMIC-IV	GB	0.848	0.250	0.656	0.230	0.3%	5,000
eICU to MIMIC-IV	MLP	0.823	0.248	0.657	0.230	0.0%	5,000

Table S13: Positive-class versus predicted-class confidence definition, all scenarios.

Scenario	Model	AUC*	C mean ($\hat{p} \to$ predicted-class)	T mean ($\hat{p} \to$ predicted-class)	Pass % ($\hat{p} \to$ predicted-class)
MIMIC-IV internal (mortality)	LR	0.862	0.111 → 0.904	0.232 → 0.708	0.0 → 83.1
	GB	0.872	0.124 → 0.902	0.262 → 0.729	2.6 → 90.7
	MLP	0.825	0.156 → 0.863	0.271 → 0.696	0.0 → 79.2
MIMIC-IV internal (prolonged)	LR	0.780	0.334 → 0.762	0.365 → 0.622	3.9 → 37.0
	GB	0.802	0.334 → 0.746	0.365 → 0.613	5.8 → 44.5
	MLP	0.753	0.335 → 0.731	0.379 → 0.617	5.5 → 52.1
eICU internal (mortality)	LR	0.819	0.095 → 0.915	0.222 → 0.714	1.1 → 87.8
	GB	0.840	0.106 → 0.906	0.229 → 0.708	0.7 → 85.9
	MLP	0.701	0.074 → 0.927	0.222 → 0.734	0.0 → 96.0
eICU internal (prolonged)	LR	0.765	0.259 → 0.771	0.321 → 0.627	0.9 → 51.8
	GB	0.761	0.261 → 0.768	0.321 → 0.625	1.1 → 40.6
	MLP	0.547	0.130 → 0.883	0.244 → 0.696	0.0 → 86.9
MIMIC-IV → eICU (mortality)	LR	0.812	0.083 → 0.924	0.215 → 0.719	0.0 → 91.6
	GB	0.807	0.181 → 0.854	0.296 → 0.700	3.2 → 78.8
	MLP	0.776	0.049 → 0.955	0.230 → 0.774	0.8 → 97.7
eICU → MIMIC-IV (mortality)	LR	0.845	0.136 → 0.891	0.248 → 0.700	1.0 → 79.3
	GB	0.842	0.198 → 0.845	0.284 → 0.672	3.1 → 68.7
	MLP	0.813	0.139 → 0.868	0.248 → 0.685	0.2 → 79.1

References

[1] Zhang, T., Kishore, V., Wu, F., Weinberger, K. Q. & Artzi, Y. BERTScore: evaluating text generation with BERT. In *International Conference on Learning Repre-*

Table S14: Linear versus nonlinear formulations, positive-class definition.

Scenario	Model	Linear pass %	Multi. Pass %	Interact pass%	Agreement L vs. M	r(L, M)
MIMIC-III→IV	LR	0.94%	0.90%	0.94%	99.96%	0.969
MIMIC-III→IV	GB	6.74%	6.32%	6.74%	99.58%	0.979
MIMIC-III→IV	MLP	3.68%	1.44%	3.68%	97.76%	0.976
MIMIC-IV→III	LR	1.62%	0.42%	1.62%	98.80%	0.960
MIMIC-IV→III	GB	6.18%	4.88%	6.18%	98.70%	0.985
MIMIC-IV→III	MLP	2.04%	1.06%	2.04%	99.02%	0.967

Table S15: S-component ablation, positive-class definition.

Scenario	Model	Full pass%	No-S pass%	Agreement	(mean)	Cases gained
MIMIC-III→IV	LR	0.94%	0.94%	100.0%	0.074	0
MIMIC-III→IV	GB	6.74%	6.72%	99.98%	0.078	1
MIMIC-III→IV	MLP	3.68%	3.68%	100.0%	0.078	0
MIMIC-IV→III	LR	1.62%	1.62%	100.0%	0.079	0
MIMIC-IV→III	GB	6.18%	4.88%	98.70%	0.080	65
MIMIC-IV→III	MLP	2.04%	2.04%	100.0%	0.084	0

sentations (ICLR) (2020). https://openreview.net/forum?id=SkeHuCVFDr
