# Supplementary material for: Governing Clinical AI Deployment: A Mathematical Framework for Transparency-Dependent Autonomy
Source: Healthcare (Basel). 2026 Nov 3;14(21):3736. doi: 10.3390/healthcare14213736 (PMC13762715; doi:10.3390/healthcare14213736)
Supplement: Supplementary file 1 [file healthcare-14-03736-s001.zip › TRIPOD_AI_checklist.pdf]

TRIPOD+AI Checklist — healthcare-4515937

Reporting guideline: Collins GS, Moons KGM, Dhiman P, et al. TRIPOD+AI statement: updated guidance for reporting clinical prediction models that use regression or machine learning methods. BMJ. 2024;385:e078378. Item locations refer to section, table, and figure numbers in the manuscript.

Section	Item	Description	Location in manuscript
Title and Abstract	1 Title	Identify study as developing/validating a prediction model; state target population and outcome.	Title; Abstract Background/Objective
	2 Abstract	Summary of objectives, data, participants, model, performance, limitations.	Abstract (Background/Methods/Results/Conclusions)
Introduction	3a Background	Clinical context and rationale.	Section 1, paragraph 1
	3b Objectives	State aims and intended use.	Section 1, final paragraph
Methods	4a Source of data	Datasets, sources, dates.	Sections 2.1, 2.7, 2.8; S1 Section 5
	4b Participants	Inclusion/exclusion, settings.	Section 2.1; S1 Section 5
	5a Outcome definition	Outcome and measurement.	Section 2.3 (prolonged ICU stay >72 h; in-hospital mortality); S1
	5b Predictors	Predictor definitions and timing (first 24 h of ICU admission).	Section 2.3; S1 Section 6
	6 Sample size	Justification of cohort and evaluation sample sizes.	Sections 2.1, 2.12 (50-case justification), 2.6 (power statement)
	7 Missing data	Handling of missing data.	Section 2.3 (train-fit median imputation; 91.7% eICU temperature imputation disclosed)
	8b Analytical pipeline	Model development pipeline, calibration procedure.	Sections 2.3 (OOF isotonic calibration; C-prime definition), 2.5; S1 Sections 7, 9 (corrected)
	9 Model development	Algorithm, hyperparameters, feature selection.	Section 2.3 (LR/GB/MLP; 36 predefined features, listed in S1 Section 14.1); S1 Section 7.1
	11 Model performance	Discrimination/calibration metrics.	Sections 2.6, 3.1, 3.5, 3.10 and Table 2 (leak-free AUCs; ECE); risk–coverage in Section 3.8
	13a Objectivity (binding)	Blinding of outcome assessment.	Limitation: physician raters were outcome-aware (Section 4, limitations; blinded redesign committed)
	13b Data leakage	Prevention of leakage into training/calibration.	Section 2.3; S1 Section 7.1.3 correction note (out-of-fold calibration)
	15 Model validation	Internal and external validation.	Sections 2.7, 2.8, 3.9, 3.10 (MIMIC-III temporal; eICU external)
	16a-b Update/fairness	Model updating; subgroup/fairness analysis.	Section 4 (limitations; weights re-optimization); subgroup analysis listed as limitation/future work
Results	17 Participants flow	Numbers at each stage.	Sections 2.1, 3.1 (5,000; 4,000/1,000; 3,500/1,500 scenario splits), Table 2
	18a-b Model performance	Performance with uncertainty.	Tables 1–4; Figures 2–4; Sections 3.1, 3.8, 3.10
	19 Model updating	Any updating during validation.	None performed (stated)

Discussion	20a-d Key results/Limitations/Interpretation/Implications	Balanced interpretation with limitations.	Sections 4 (incl. expanded limitations on E, S, clinician study design), 5
Other	22 Supplementary information	Availability of protocol, code, full analysis.	Data Availability; Code deposit (DOI at submission); Supplementary Files S1 and S2
	23 Funding	Funding sources and role.	Funding statement
